# On the possibility of using waste disposable gloves as recycled fibers in sustainable 3D concrete printing using different additives

**DOI:** 10.1038/s41598-023-37803-9

**Published:** 2023-07-04

**Authors:** Seyed Sina Mousavi, Mehdi Dehestani

**Affiliations:** Faculty of Civil Engineering, Babol Noshirvani University of Technology, Postal Box: 484, Babol, 47148-71167 Iran

**Keywords:** Engineering, Civil engineering

## Abstract

Due to the Covid-19 pandemic, using large amounts of personal protective equipment (PPE) throughout the world has extensively increased in recent years. The lack of a practical method to dispose of these recycled materials is one of the main concerns of researchers. Hence, comprehensive experimental tests were conducted in the present study to investigate the feasibility of using disposable gloves in mortars to achieve a sustainable mixture. Accordingly, latex and vinyl gloves as recycled fibers were considered in the experimental program to improve the sustainability of 3D printing concrete. As using these recycled materials causes some deficiencies for printing layers, different mineral and chemical admixtures were used in the present study, including graphene oxide nanomaterials, polyvinyl alcohol, Cloisite 15A nanoclay, and micro silica fume. Also, the hybrid use of latex, vinyl, and polypropylene (PP) fiber was considered to improve the printability of concrete mixtures containing waste fibers. Moreover, the effect of internal reinforcement was also considered by using plain steel wire mesh to increase the composite behavior of printed layers in this simplified experimental program. Results indicate that the synergic influence of recycled fibers and admixtures meaningfully enhanced the 3D printing properties of mortar so that about 20%, 80%, 50%, and more than 100% improvements were obtained for workability, direct tensile strength, flexural strength, and buildability index respectively. However, an average percentage − 28.3% reduction was recorded for the concrete compressive strength. Sustainability analysis also showed that using waste disposable gloves considerably reduced CO_2_ emissions.

## Introduction

With the spread of the COVID-19 virus worldwide, disposable gloves have been considered as personal protective equipment (PPE) more than ever in the past few years^1^. In addition, different health and public places use disposable gloves, including hospitals, scientific laboratories, manufacturing facilities, halls, and museums. The growing use of disposable protective gloves and discarding them in random places may alarmingly cause a significant increase in environmental pollution. Different types of disposable gloves were produced by companies, such as latex, vinyl, and nitrile gloves. Natural rubbers, synthetic rubbers, polyethylene plastic, and polyvinyl chloride plastics are the main materials of these disposable gloves. Nitrile and vinyl gloves are produced from synthetic materials, while natural latex rubber is the main component of latex gloves. The mechanical strength of nitrile gloves is greater than vinyl ones. Polyvinyl chloride, a petroleum-based film, is the main component of vinyl gloves. Although latex gloves produced by natural rubber are very stretchy, the issue of being allergic to latex gloves for many people results in high consumption of disposable vinyl gloves. However, still, in chemical and medical scientific laboratories, researchers widely use latex during their work to shield their skin against dangerous materials. Accordingly, there is a significant amount of disposable gloves accumulated in the world, and it will be a costly and time-consuming process to return them to the reuse cycle as gloves. For instance, Marandi^2^ reported that around 5000 tons of latex gloves are consumed in Iran yearly, which was published in 2006 without considering COVID-19. The occurrence of the COVID-19 pandemic has aggravated this dilemma. Accordingly, a practical solution is necessary to return this huge waste material depot to the use (non-medical) cycle in a healthy and non-contaminated process. In addition to the method of reproducing gloves from contaminated waste, which is very expensive and is not a practical solution, the issue of burying these contaminated gloves is also not practical and will have potential risks to the environment^3,4^. In this research area, Shu et al.^5^ highlighted that landfilling rubber waste in large volumes could no longer be interrelated with increased land prices; accordingly, disposal of rubber waste becomes an expensive way. Moreover, during latex gloves production, a highly cross-linked thermoset structure of rubber material over the vulcanization procedure is pursued by companies to attain great resistance properties against acids, alkali, and chemical solutions. This results in difficulties in degrading natural rubber (from latex gloves) in the environment^6,7^.

One of the effective techniques to solve the issue of waste disposal is using them as additives within concrete mixtures^8^. Adding waste materials within construction industries can be an approving solution to decrease greenhouse gas emissions and environmental contaminations^9^. Correspondingly, researchers recommended using waste rubber^10–14^ and plastic^15–17^ in concrete construction. In this way, it can be deduced from the literature that disposable gloves can also be similarly used as additives in concrete due to their accessibility and low costs, taking into account some limitations regarding their dosage and contaminations^18–20^. Also, the good elastic properties of waste disposable gloves can be helpful for different applications, such as damping mechanisms, thermal insulators, and noise insulators^4^. In this context, previous studies confirmed that adding waste gloves enhanced the mechanical properties^6,21–23^.

Although extensive research concentrated on using waste rubber^24–26^ and polyvinyl chloride (PVC)^27–32^ within concrete mixtures, limited studies worked on using waste gloves as admixtures (powders, fine aggregates, and recycled fibers) within concrete mixtures. In this field, Mortazavi et al.^33^ reported that adding waste latex gloves (5%) improved the ductility of modified bitumen. Followed by their research, ALbiajawi et al.^18^ conducted an experimental test to determine the influence of ground latex gloves as coarse aggregate in concrete. Based on their results, using waste latex gloves along with silicone catheters importantly improves concrete water absorption. Additionally, their results indicate that using 10% waste latex gloves causes a high reduction in the concrete compressive strength. This strength reduction was similarly confirmed in the recently published experimental work by Mousavi and Dehestani^19^, where they used waste latex and vinyl gloves as recycled fibers within mortars prepared for concrete 3D printing. They also used graphene oxide (GO) nanomaterials to compensate for this strength reduction. Despite these valuable and novel findings, there is a considerable research gap in this field, which requires more investigations for future studies.

**Figure 1:**
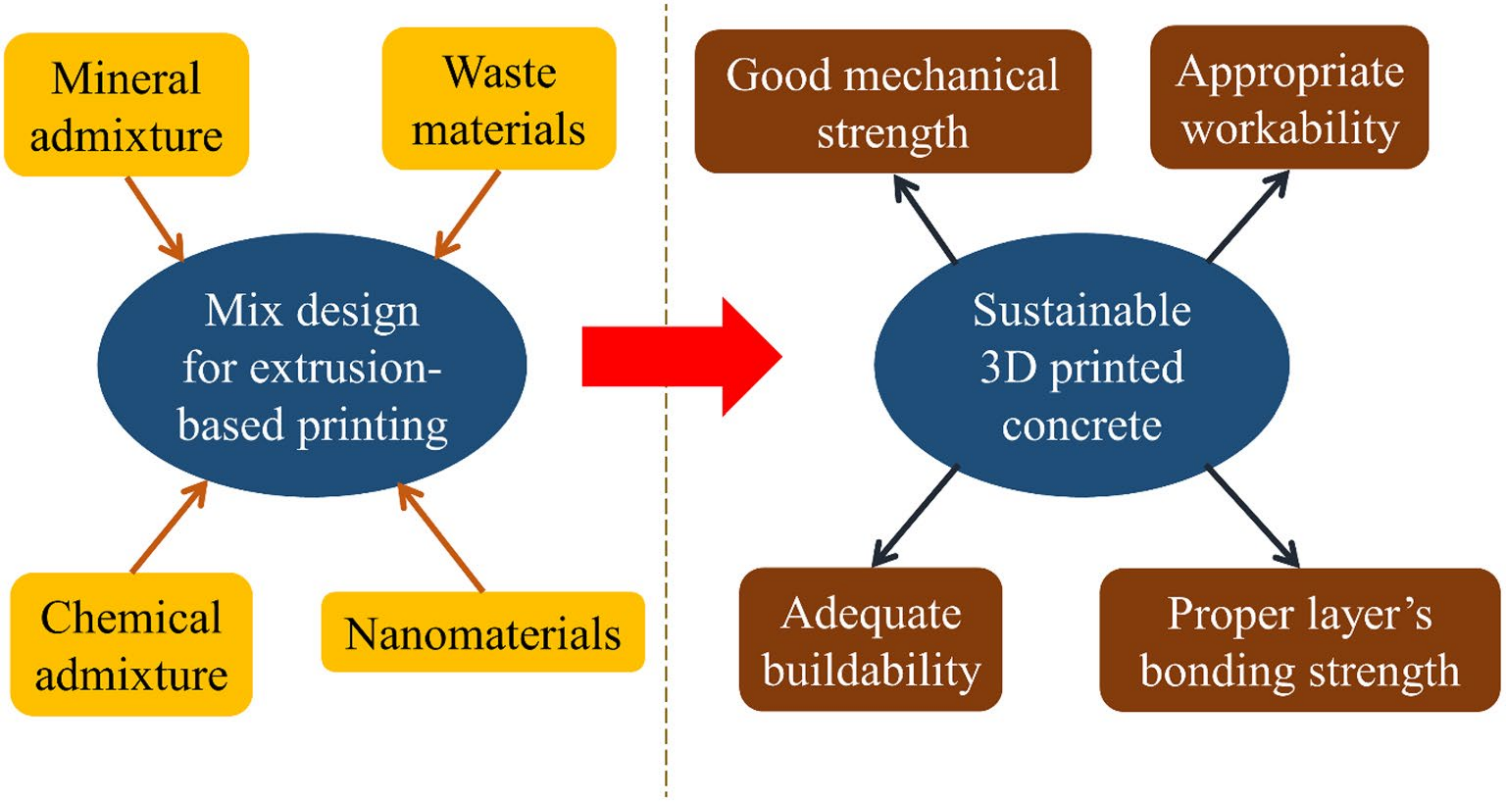
Main objective of the present study.

Regarding three-dimensional concrete printing (3DCP), many studies recently concentrated on using new mixtures to improve the sustainability of compendious composites^34,35^. 3DCP method considerably improved concrete sustainability by reducing construction cost in the construction industry, improving the feasibility of designing various construction forms, reducing the number of moulds needed for construction, managing the number of materials consumed to reduce material waste, and the number of workers needed^36,37^. However, as shown in Fig. 1, appropriate fresh and hardened properties should be provided for a mixture considered for the 3DCP method. To achieve this specific mixture, non-economic and unsustainable production and consumption of some special materials are required, which results in an important concern regarding the sustainability of the 3DCP method, such as using a high content of microfibers (at least 2.0%) in ultra-high ductility concrete (UHDC) and different types and dosages of chemical admixtures. Working on novel mixture composition plays a major role in controlling this issue in the 3DCP method^36,38^. Accordingly, previous studies have been continuously working in recent years to use waste and recycled materials in the 3DCP method^35,39,40^.

Following these researchers, Mousavi and Dehestani^19^ recently worked on new aspects of using waster materials in the 3DCP method, which is sustainable mortars using recycled fibers from waste disposable gloves. They found that recycled fibers can efficiently improve the sustainability of cementitious composites needed for 3DCP. However, their findings show that there are also some challenges regarding shape stability and printability of mixtures containing latex and vinyl recycled fibers. They recommended that using nanomaterials can partially compensate for this deficiency, which was similarly confirmed by the literature^41,42^. Since a significant amount of waste disposable gloves have accumulated worldwide and reusing them to produce new gloves is complicated, it is necessary to have a cheap and accessible solution for the sustainable use of these waste materials. Accordingly, it is essential to achieve a practical solution for compensating the deficiencies of 3DCP using latex and vinyl gloves as recycled fibers. Since the recently published paper by Mousavi and Dehestani^19^ was one of the pioneers of a detailed study on this type of 3DCP, many additives should be investigated to overcome the deficits of latex- or vinyl-contained 3DCP. Different scenarios can be proposed to improve the printing properties of recycled fibers-contained 3DCP, including (1) using supplementary cementitious materials (SCMs); (2) using nanomaterials; (3) using chemical additives such as polymers; (4) using hybrid fibers; and (5) considering internal reinforcement between layers. Hence, to address the observed challenges in the 3DCP mixtures containing recycled fibers, the present study aims to comprehensively extend the findings of Mousavi and Dehestani^19^ and follow specific objectives as follows:

1. How much does using chemical additives affect the fresh and mechanical characteristics of the mortar containing recycled latex and vinyl fibers?
2. How do micromineral additives impact the 3D printing characteristics of mortars using latex and vinyl fibers?
3. What is the influence of nanomaterials on the 3D printing characteristics of mortars using latex and vinyl fibers?
4. What is the synergistic effect of the polypropylene (PP)/latex and latex/vinyl hybrid fibers on the 3D printing properties of cementitious composites?
5. How much welded wire mesh (WWM) as internal reinforcement efficiently enhances bond strength between layers in mixtures containing waste latex gloves with vinyl ones?

To this end, an extensive experimental program was carried out in the present investigation. Different chemical and mineral admixtures were also considered in the present study to improve the fresh and mechanical properties of mortars containing latex and vinyl gloves, including graphene oxide nanomaterials (GO), polyvinyl alcohol (PVA), clay mineral, and micro silica fume. Flow table and shape stability tests were implemented to measure the workability and buildability properties of mixtures. Hardened density, compression test, direct tensile test, and bending tests were implemented in the present study regarding hardened characteristics. Recycled fibers were produced by using waste latex and disposable vinyl gloves. Additionally, welded wire mesh (WWM) was also used to increase the bond strength between layers of sustainable 3DCP samples exposed to lateral loading. Moreover, Statistical analysis was considered in each property to recognize the most noteworthy parameters affecting the performance of the proposed mixtures.

## Experimental program

### Materials and mixtures

**Figure 2:**
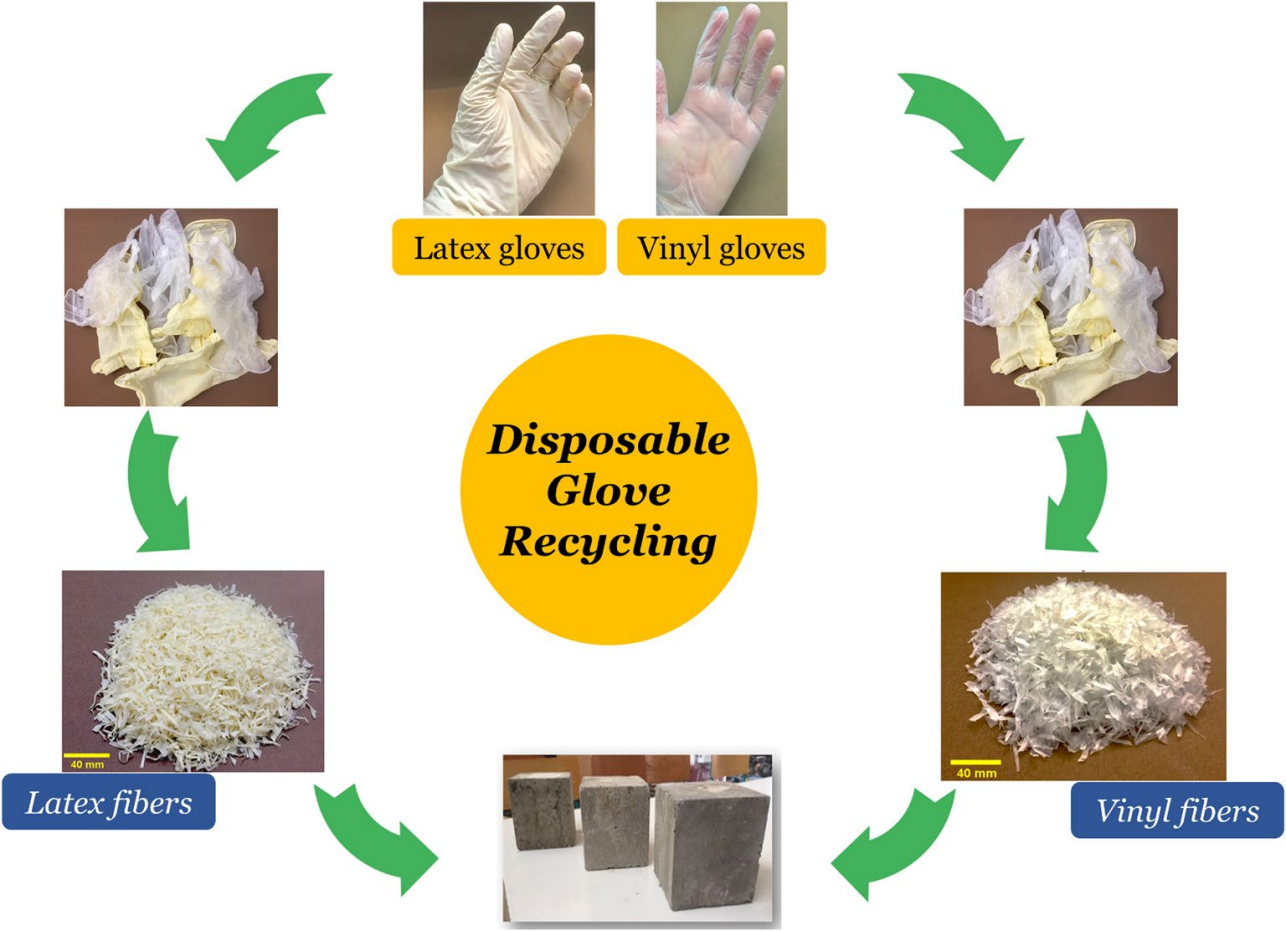
Recycled fibers used in the present study from waste latex and vinyl gloves.

**Figure 3:**
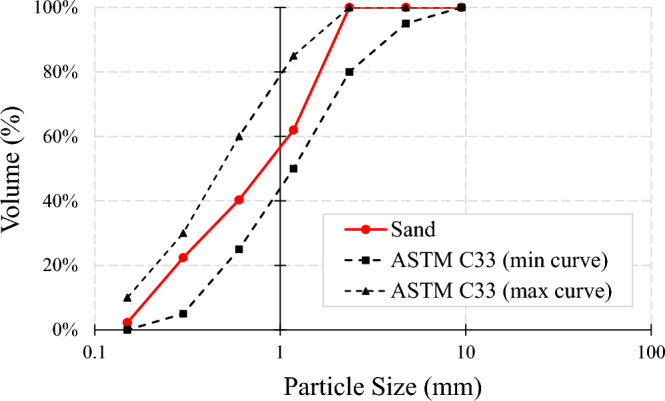
Particle size distribution curve of sand used in the present study.

**Figure 4:**
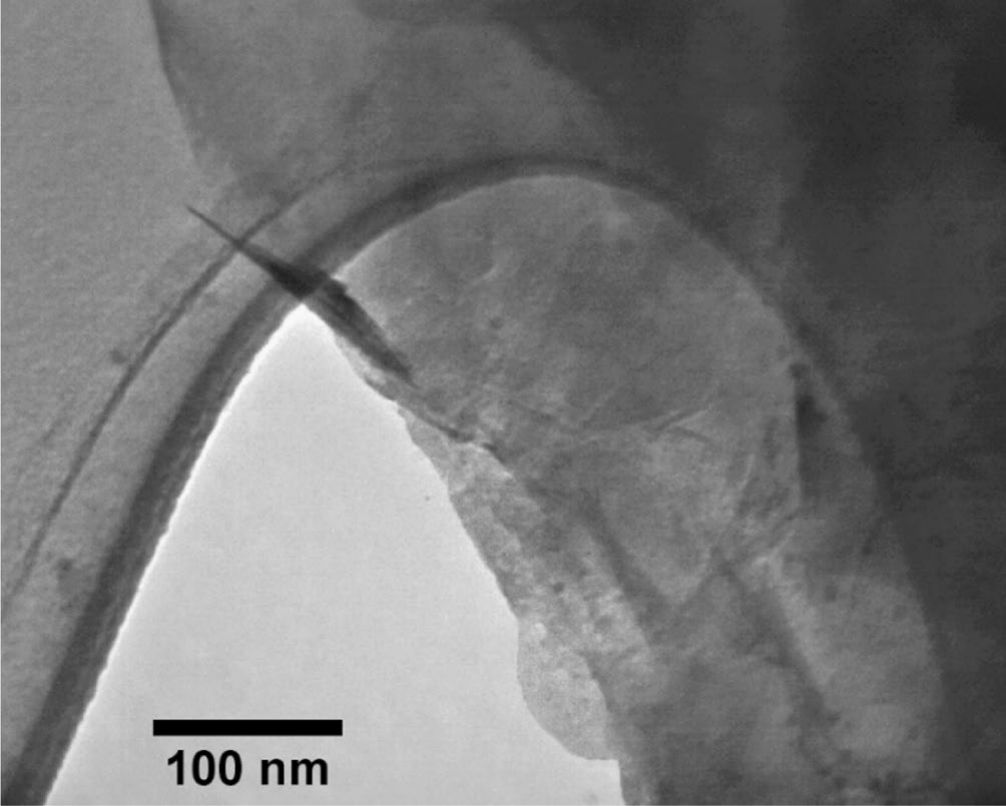
TEM image of graphene oxide (GO) nanomaterials considered in the present study.

**Figure 5:**
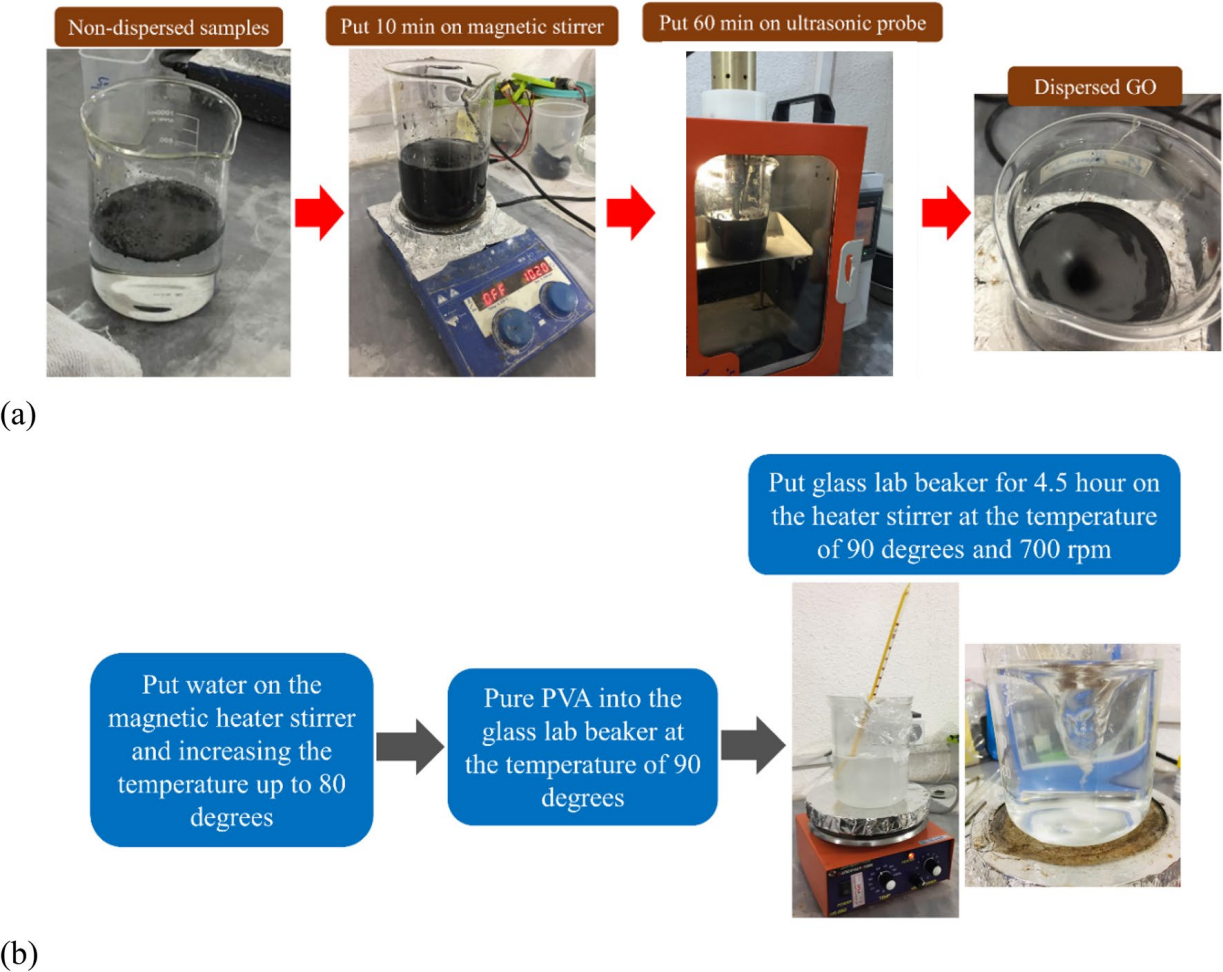
Preparation of admixtures: (**a**) GO dispersion process; (**b**) PVA dispersion process.

Type II Portland cement with a density of 3.1 g/cm^3^ was used in this study. As explained in Table 1, eighteen mixtures were considered for the experimental program, with a constant water-to-binder ratio of 0.42. As shown in Fig. 2, latex and vinyl recycled fibers were produced from waste disposable gloves with different volume fractions of 0.1% and 0.2%, with an average length and diameter of 16 mm and 2.0 mm, respectively. The fiber preparation process was performed through a hand-making process without a particular device, including cleaning the surface of the gloves and cutting the fibers from the disposable gloves. After the preparation process, the fibers of the same size were selected for the concrete mixtures. A maximum size of 2.36 mm was selected for the fine aggregate with a sand-to-cement ratio of 1.5 for producing the appropriate mixture needed for the 3DCP method. The particle size distribution curve of sand used in the present study is shown in Fig. 3. Different chemical and mineral additives along with polypropylene (PP) fiber were considered in the mixtures (Table 1), including silica fume (SF), nanoclay (C), graphene oxide (GO), and polyvinyl alcohol (PVA) powder. The dosage of 0.03% (by weight of cement) was selected for GO, which was similarly tested by Bhojaraju et al.^43^. GO nanomaterials had a specific surface area of 40 m^2^/g, purity of 98.5%, average flake thickness of 60 nm, and particle (lateral) size of less than 7 µm. Figure 4 shows the TEM image of GO. The GO dispersion process is explained in Fig. 5a, where a proper dispersion technique was considered before using GO within the mixture, which was comprehensively explained in the recent paper by Mousavi and Dehestani^19^. It is worth mentioning that reference mixtures tested by Mousavi and Dehestani^19^ were also used in the present study to compare the results and determine the efficiency of chemical and mineral additives within 3D printable concrete. Nanoclay (Cloisite 15 A) with a modified concentration of 125 meq/100 g clay was also used to improve the mixture's buildability and control the extra water within the mixture. The organic modifier of nanoclay was dimethyl, dehydrogenated tallow, and quaternary ammonium. The moisture content of nanoclay was less than 2.0%. X-ray diffraction d-spacing of nanoclay was 31.5 Angstroms. Cloisite 15 A nanoclay has a 1.66 g/cm^3^ specific density and 0.1729 g/cm^3^ bulk density. This type of clay has a smaller than 2.0 μm particle size. Hereafter, factor “C” is used to show the results of mixtures containing Cloisite 15A nanoclay. A replacement dosage of 2.0% (by weight of cement) was selected for nanoclay. To adjust the mixture buildability, PVA powder was also considered in the experimental program with the chemical formula of (C_2_H_4_O)_n_. PVA has a density of 1.19 g/cm^3^ and an ignition temperature of 450 °C. As shown in Fig. 5b, to disperse PVA within the water of mortar, at first, a container with mixing water was put on a magnetic heater stirrer with increasing temperature up to 80°. Then, PVA was mixed with water, and the temperature was increased to 90°. The glass lab beaker was kept in this condition for 4.5 h. at the temperature of 90° and 700 rpm. Finally, the dispersed container cooled at laboratory temperature, and the water content was checked. Commercial micro silica fume (SF) was also used in some mixtures to improve the mixture's consistency and mechanical strength. As mentioned in Table 1, a replacement dosage of 10% (by weight of cement) was selected for SF. It is worth mentioning that no superplasticizer was added to the mixtures. PP fiber was also used in one mixture (0.1Latex + 0.1PP) to determine the effect of hybrid fibers on the results. PP fibers had a density of 0.91 g/cm^3^, elongation of 80%, and tensile strength of 400 MPa. After demolding, specimens were cured in a water tank maintained at 20 °C and 60% RH for a 28-day curing period.

**Table 1:** Mix proportions of mortars (kg/m^3^).

Mixes	Cement	Water	Sand	SF	C	GO	PVA	Latex fiber	Vinyl fiber	PP fiber
RF	450	189	675	–	–	–	–	–	–	–
0.1Latex	450	189	675	–	–	–	–	1.02	–	–
0.2Latex	450	189	675	–	–	–	–	2.04	–	–
0.2Vinyl	450	189	675	–	–	–	–	–	2.2	–
0.1Latex + GO	449.86	189	675	–	–	0.135	–	1.02	–	–
0.2Latex + GO	449.86	189	675	–	–	0.135	–	2.04	–	–
0.2Vinyl + GO	449.86	189	675	–	–	0.135	–	–	2.2	–
0.1Latex + C	441	189	675	–	9	–	–	1.02	–	–
0.1Latex + PVA	450	189	675	–	–	–	2.7	1.02	–	–
0.2Latex + PVA	450	189	675	–	–	–	2.7	2.04	–	–
0.2Latex + GO + C	440.78	189	675	–	9	0.135	–	2.04	–	–
RF + SF	405	189	675	45	–	–	–	–	–	–
0.1Latex + SF	405	189	675	45	–	–	–	1.02	–	–
0.2Latex + SF + GO	404.86	189	675	45	–	0.135	–	2.04	–	–
0.1Latex + 0.1Vinyl	450	189	675	–	–	–	–	1.02	1.1	–
0.1Latex + 0.1PP	450	189	675	–	–	–	–	1.02	–	0.92
0.2Latex + 0.2Vinyl	450	189	675	–	–	–	–	2.04	2.2	–
0.2Latex + 0.2Vinyl + GO	449.86	189	675	–	–	0.135	–	2.04	2.2	–

*GO* graphene oxide, *C* Cloisite 15A nanoclay, *SF* (*MS*) micro silica fume, *PVA* polyvinyl alcohol, *PP* polypropylene.

### Experiment design

**Figure 6:**
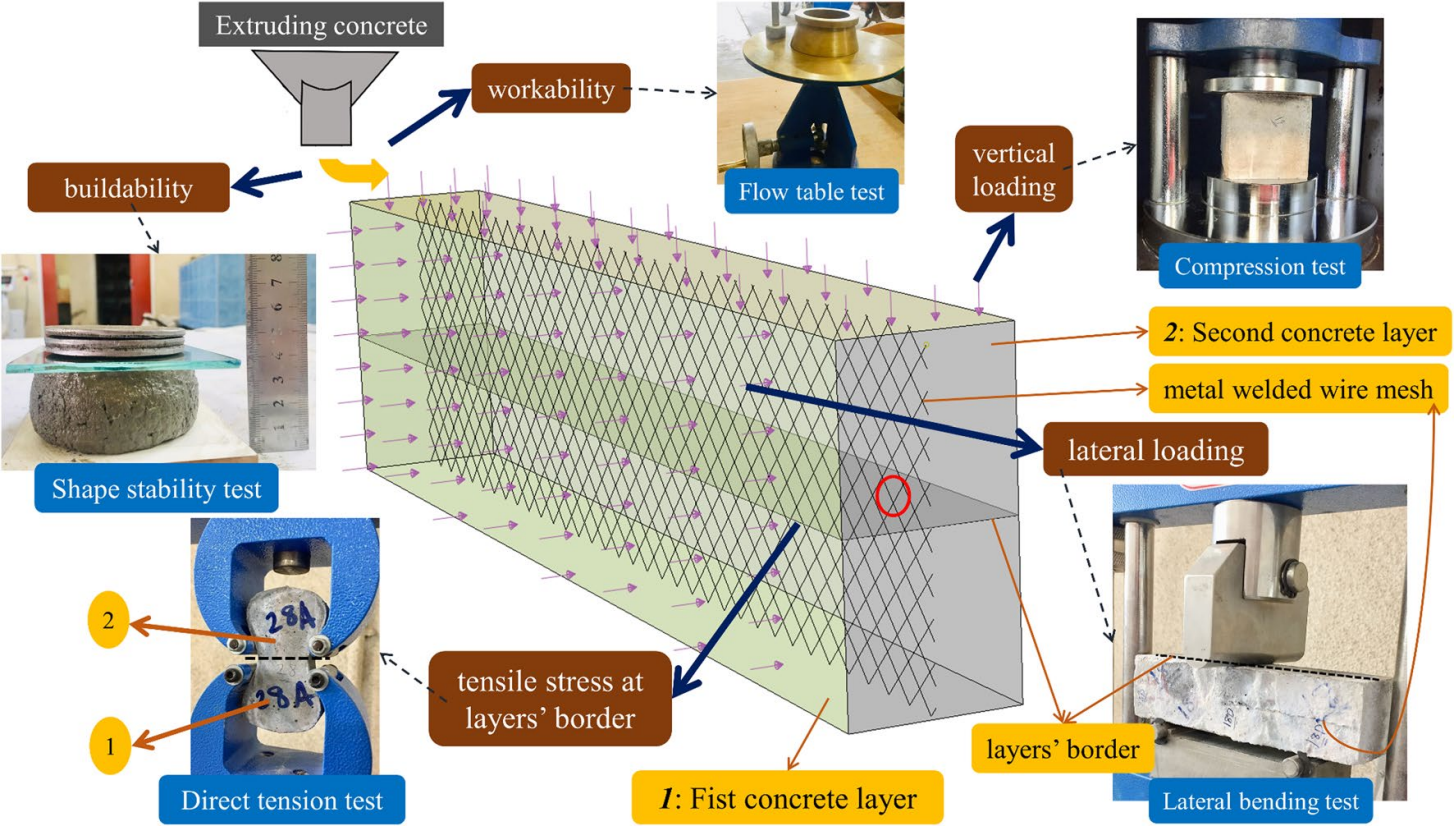
Experimental tests considered in the present study.

As shown in Fig. 6, five different tests were considered for the experimental program to (1) determine the probability of using recycled fibers with admixtures on fresh properties of mortar appropriate for 3DCP; and (2) investigate the effect of chemical and mineral admixtures on the mechanical characteristics of mixtures containing recycled latex and vinyl fibers. To determine the mixture's workability, the spread diameter was measured by the flow table test based on ASTM C1437^44^. Also, a shape stability test was performed to check the mixture buildability, which was recommended by the literature^42,45–47^. This test evaluates the instant axial deformation of the fresh mixture under progressively increasing axial loading loads (by applying weights). This test shows the stability and buildability of layers to withstand the weights of upper layers without any collapse. Regarding mechanical properties, 50 mm cubes and briquet specimens were considered in the present study to determine the compressive strength and direct tension (ASTM C190^48^) strength of mortars. Moreover, to indirectly study the bond behavior between mortar layers, the three-point lateral bending test was performed after 28 days of curing. Two discrete mortar layers considered in this simplified bending test were poured separately within a 40 × 40 × 160 mm prism mould with a delay of 10 min. Moreover, to improve the consistency between printed layers, internal reinforcement by rhombus-shaped metal welded wire mesh (WWM) with a thickness of 0.50 mm and dimensions of 4.67 × 2.3 mm was used in the experimental specimens. WWM had yield and ultimate strengths of 400 MPa and 600 MPa, respectively. A comparison study was conducted to determine the effect of using WWM in sustainable mortars containing recycled fibers and admixtures. As shown in Fig. 6, lateral loading was applied to the reinforced-prism specimens to determine the of WWM on maximum bending capacity and crack propagation throughout the layers.

## Results

### Workability

**Figure 7:**
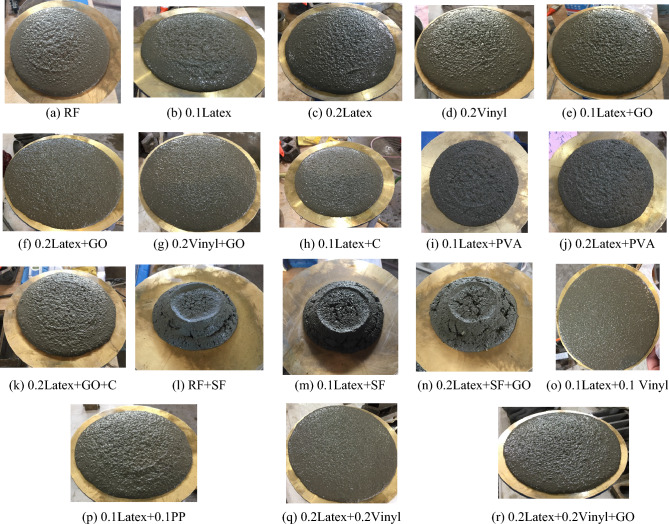
Spread diameter of mixtures after flow table test.

**Figure 8:**
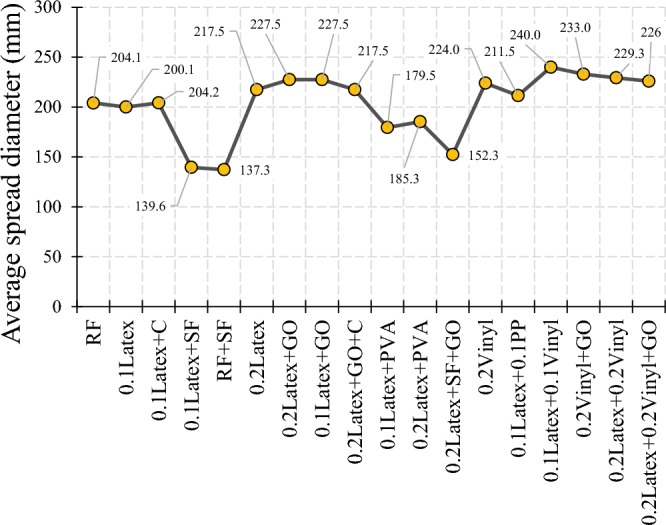
Results of workability from flow table test.

**Figure 9:**
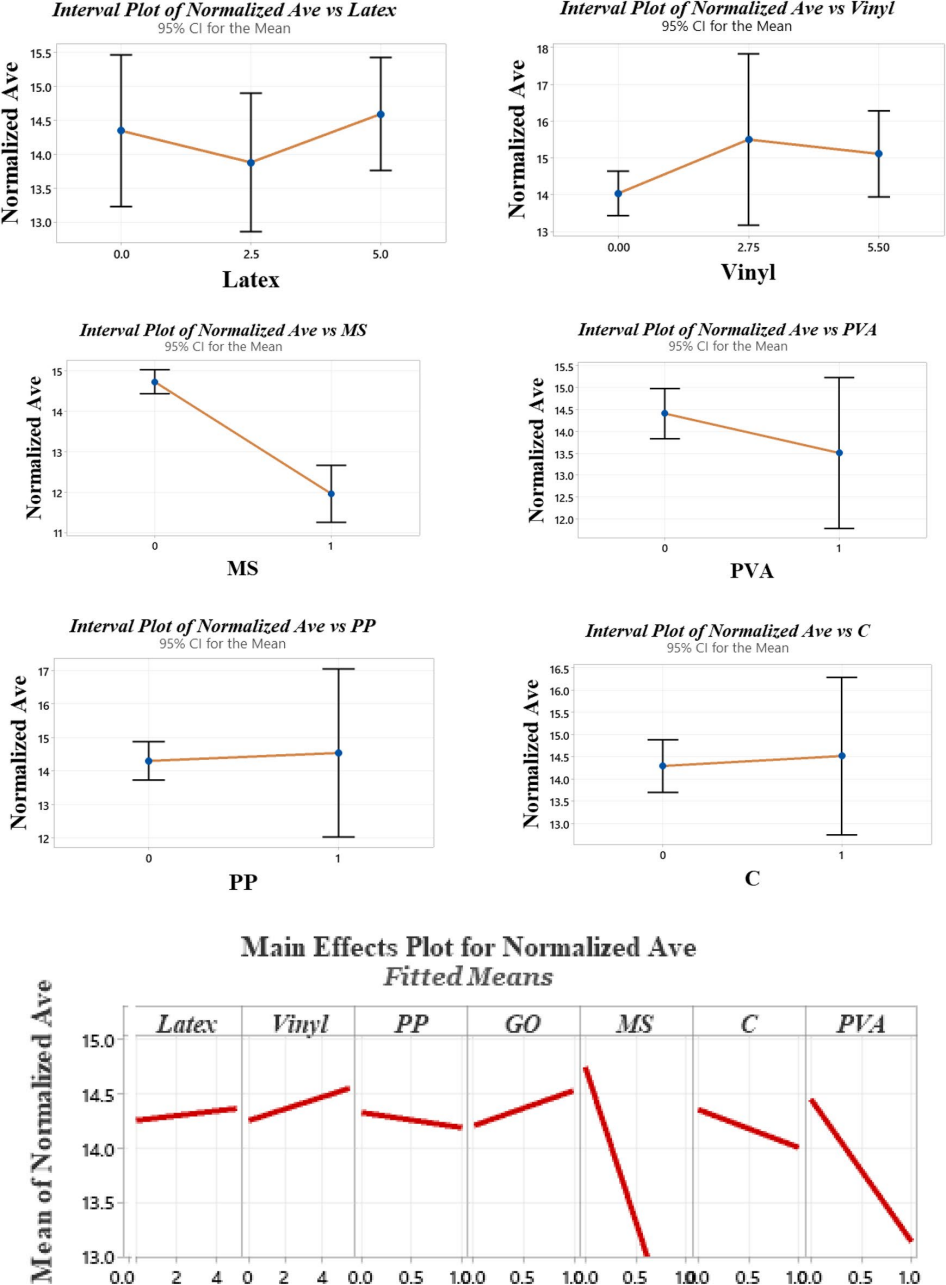
Analysis of variance (ANOVA) for workability results.

Generally, the literature recommended using mixtures with a high quantity of fibers for use in 3DCP, such as ultra-high ductile concrete (UHDC)^42,49^. This high content of fibers generates serious issues for workability. For instance, the findings of Ye et al. revealed that using a high amount of fibers substantially reduces the spread flow diameter^49^. Hence, using mineral and chemical admixtures can compensate for this issue. The spread diameters of mixtures after the flow table test are shown in Fig. 7. The results of the flow table test are depicted in Fig. 8. Spread diameter of 204.1 mm was considered for the reference mortar (RF) without using a superplasticizer. No additional water was used for the mixture to precisely monitor the workability reduction in mixtures. Overall findings show that using recycled fibers produced by disposable gloves causes a promising impact on concrete workability, so that the mixture “0.1Latex + 0.1Vinyl” has the highest workability with a 17.6% improvement in mortar workability. However, the mixture “RF + SF” has the lowest workability with a 32.7% spread diameter reduction. Results indicate that using a low dosage of latex fibers does not affect the spread flow diameter, while the addition of 0.2% latex fibers results in a 6.6% improvement. In the same way, the addition of 0.2% vinyl fibers causes a 9.8% higher spread flow diameter as compared to the reference mortar. These observations may be attributed to the nature of natural rubber and polyvinyl chloride plastics. Although previous studies reported that using nanomaterials reduces concrete workability^43,50^, the results of the present study showed that the addition of GO nanomaterials causes 13.7% and 4.6% improvements on the mortar spread flow diameter in “0.1Latex + GO” and “0.2Latex + GO” mixtures, respectively. Additionally, the mortar workability of “0.2Vinyl + GO” mixture is around 4.0% greater as compared to “0.2Vinyl” mixture. To comprehensively compare the results, an analysis of variance (ANOVA) was performed by using Minitab statistical software^51^. ANOVA results are summarized in Table 2 and Fig. 9. Statistical results show that SF and PVA have the lowest P-values, which correspond to the highest impacts on the mortar workability (Fig. 9). Moreover, it can be deduced from the statistical results that latex fibers, vinyl fibers, and GO nanomaterials increase the workability. Among them, results show that GO has a higher influence as compared to vinyl fibers. Furthermore, results show that nanoclay and PP reduce workability due to the water absorption on the surfaces. Among the studied parameters, analysis shows that SF significantly reduces the mortar workability. Generally, results show that vinyl fiber has higher promising effect on mortar workability as compared to latex fiber. Results also indicate that reduction impact of PVA on workability is lower for mortar containing higher dosage of latex fiber (0.2%) as compared to 0.1% (Fig. 8). Results indicate that using hybrid fibers considerably improves the mortar workability. Among mixtures containing hybrid fibers, mixture containing 0.1% latex and vinyl fibers showed the highest workability. Compared to PP fibers, findings confirm that these recycled fibers can be used to produce 3D printing mortar with the improved workability, which is necessary for extrusion-based printing techniques.

**Table 2:** Analysis of variance (ANOVA) for workability results.

Source	DF	Adj SS	Adj MS	F-value	P-value	VIF
Latex	1	0.0265	0.0265	0.23	0.644	1.49
Vinyl	1	0.2102	0.2102	1.79	0.208	1.35
PP	1	0.0161	0.0161	0.14	0.719	1.14
GO	1	0.3499	0.3499	2.98	0.112	1.32
MS (SF)	1	15.8684	15.8684	134.96	0.000	1.29
C	1	0.1786	0.1786	1.52	0.243	1.23
PVA	1	2.4144	2.4144	20.54	0.001	1.28

### Buildability

**Figure 10:**
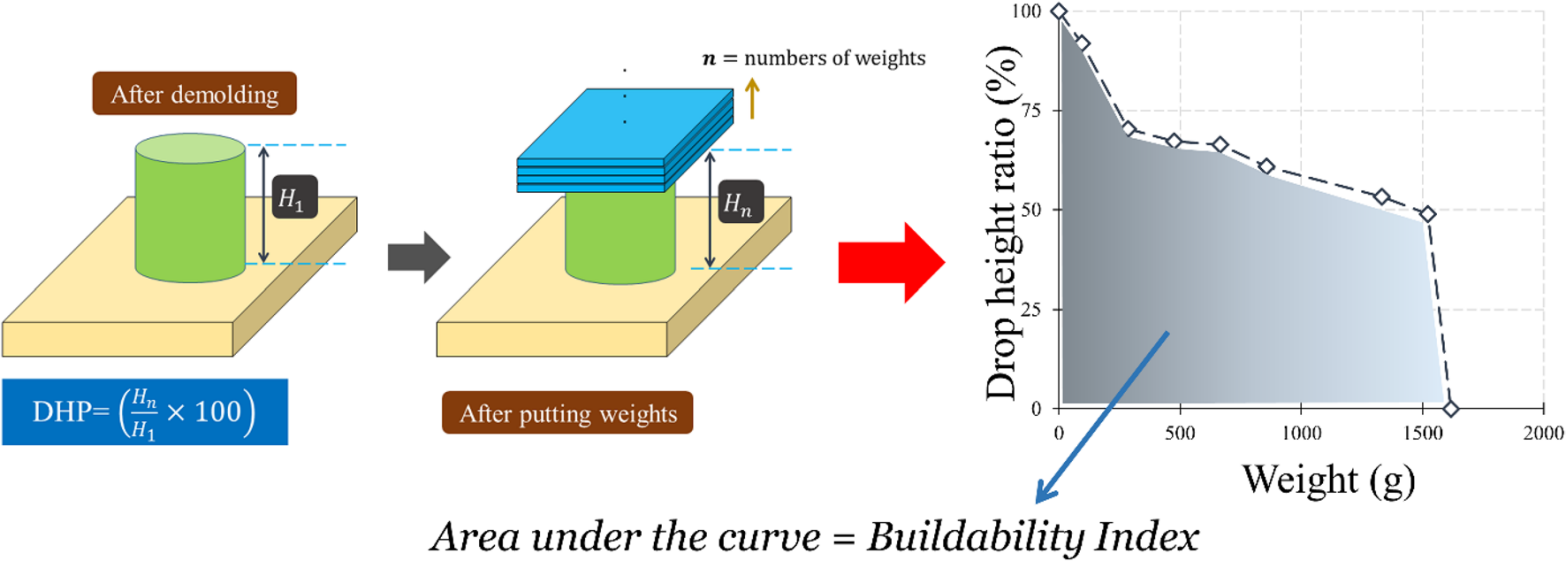
Configuration of the shape stability test.

**Figure 11:**
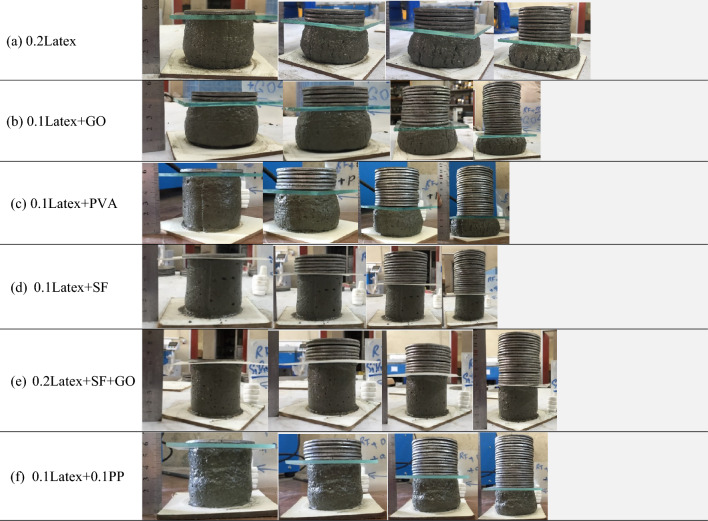
Shape stability of some mixtures after the flow table test.

**Figure 12:**
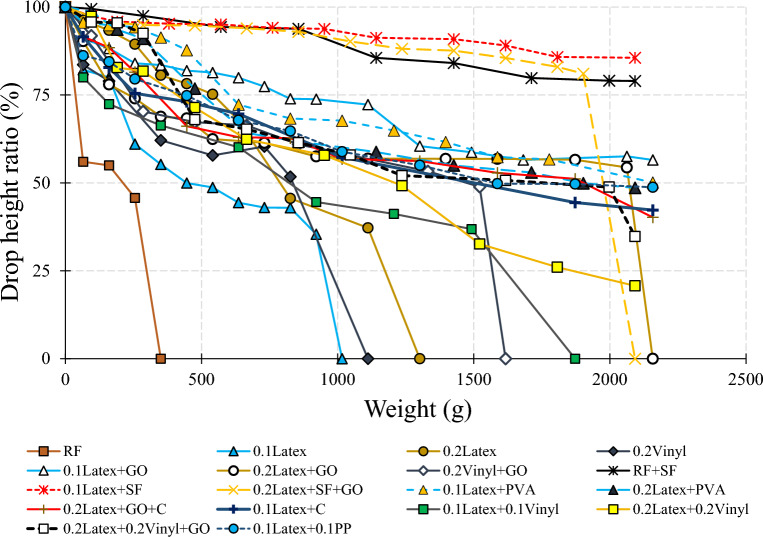
Results of the buildability test.

**Figure 13:**
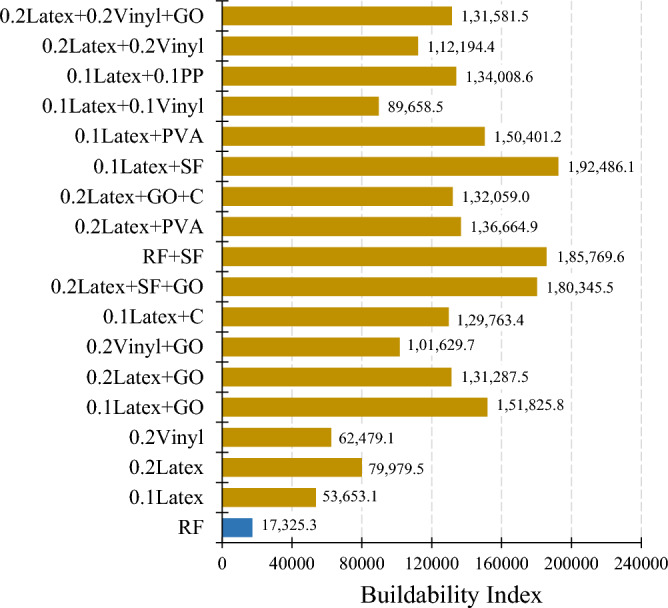
Results of the buildability index for all mixtures.

**Figure 14:**
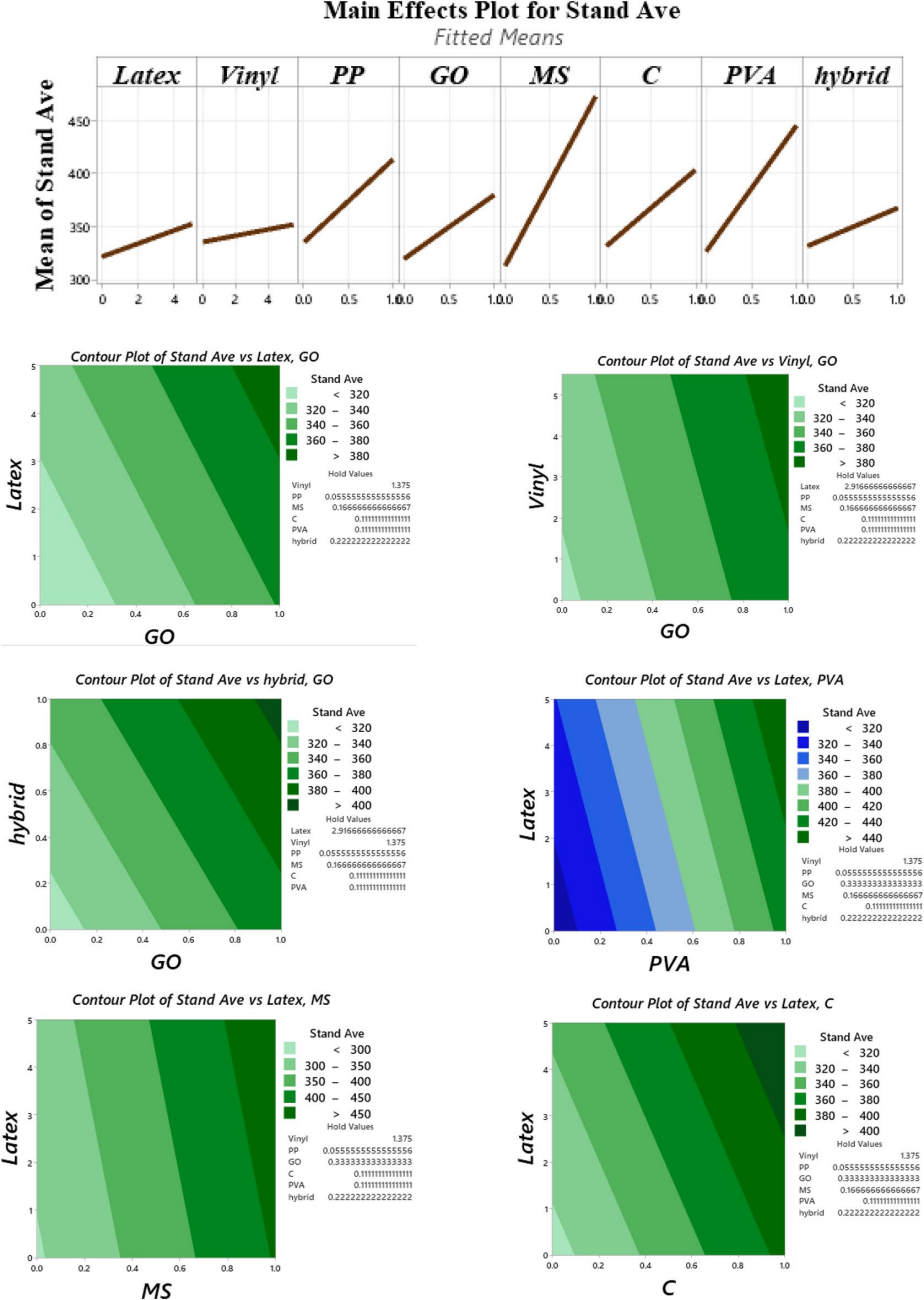
Analysis of variance (ANOVA) for shape stability test results.

A cylindrical 60 × 60 mm mould was considered to study the buildability of mixtures (Fig. 10). Factor of “drop height percentage (DHP)” is used to compare the results. This test is also well-known in this field of research as a “shape stability” test. After demolding, different steel plates (weight of 95.1 g) were put on top of the fresh samples, and the height of the samples was recorded. Accordingly, DHP is defined as the ratio of the sample height after demolding (*H*_*n*_) to the after putting weights (*H*_*1*_). A high value of DHP corresponds to a higher mixture buildability. A low value of DHP shows the weak proficiency of lower layers in supporting the weights of upper layers. This may be attributed to the low viscosity of mixtures. Two repetitions were considered for each mixture. The best performance was considered in this section. The process of the shape stability test for some mixtures is illustrated in Fig. 11. Also, measured DHP values for all mixtures versus weights of plates are illustrated in Fig. 12. The area under the DHP-weight curve was also calculated to determine the “Buildability Index”, which is illustrated in Fig. 13. Overall, findings revealed that the reference mixture (RF) has weak shape stability among other mixtures, in such a way that this mixture could not stand the weights greater than 400 g (Fig. 13). It means that premature collapse will be happened during 3D printing by using the “RF” mixture. Observations show that using recycled fibers produced by the waste disposable gloves significantly improves the 50% DHP and buildability index. For instance, “0.1Latex” and “0.2Latex” mixtures enhanced the buildability index by 209.7% and 361.6%, respectively. Correspondingly, findings show that a 260.6% improvement was obtained for the buildability index of mixtures containing 0.2% vinyl fiber. It can be deduced from the observations that latex fiber-contained mixtures have a higher buildability index than vinyl fiber ones. Findings also show that using GO nanomaterials significantly enhanced the buildability index of mixtures containing recycled fibers, which was similarly shown for normal concrete by previous studies^43^. Also, as mentioned similarly for mixtures without GO, the buildability index of latex-contained mixtures using GO is greater than vinyl/GO-contained mixtures. Overall results revealed that vinyl fiber improves the flowability factor while causing a reduction in the concrete viscosity. Accordingly, it can be deduced from the trend that vinyl-contained mixtures need nanomaterials to compensate for the buildability reduction. Moreover, results show that mixture “0.1Latex + SF” has the highest buildability index among all mixtures. To comprehensively analyze the results, an ANOVA analysis was conducted, which is mentioned in Table 3 and Fig. 14. Statistical results show that SF (or MS) has the most crucial impact on the buildability index so the lowest P-value was obtained (0.004). Similarly, Srinivas et al.^52^ found that adding SF as cement replacement enhanced the buildability of 3D printing mixtures due to higher static yield stress. In addition to the SF (or MS), the PVA has also a significant impact on the results with a P-value of 0.045. statistical results shown in Fig. 14 indicate that despite PP fiber, latex and vinyl fibers increase the mixture's buildability. Comparing the results presented in Figs. 8 and 13 show that recycled fibers have a promising effect on both the workability and buildability characteristics of the mixtures, which is very promising. Adjusting these two properties is difficult for mortar so most researchers have been using chemical admixture (mostly superplasticizers) to control concrete fluidity and viscosity. However, the findings of the present study show that by using recycled fiber, the viscosity of mixtures can be controlled without using expensive commercial admixtures. Results show that the addition of PP fiber although reduces the workability, has a positive impact on the buildability of the mixture. As shown in Fig. 14, nanoclay also improves the mixture's buildability. Similarly, Panda et al.^53^ reported that using nanoclay has a promising effect on the mixture printability in high-volume fly ash 3D printing mixtures. They attributed this observation to the enhanced thixotropic property nanoclay-contained mixtures, including high flocculation strength and yield stress. Results presented in Fig. 14 also demonstrate that the combination of recycled fibers was also helpful for improving the buildability index so that even with 0.4% recycled fibers (“0.2Latex + 0.2Vinyl” mixture) 64.5% improvement in mixture buildability was found as compared to “0.2Latex” mixture. Results also indicate that although 0.2% latex has more effective in increasing the buildability index as compared to 0.1% for normal mixtures, the synergic effect of GO with 0.1% was recorded by the experimental results. Furthermore, results also depict that in the mixture containing GO nanomaterials, latex fibers are more efficient as compared to vinyl fibers. Findings show that the PVA has a higher impact on the buildability index as compared to nanoclay and GO nanomaterials.

**Table 3:** Analysis of variance (ANOVA) for shape stability results.

Source	DF	Adj SS	Adj MS	F-value	P-value	VIF
Regression	8	73,642	9205.3	2.91	0.066	–
Latex	1	1308	1307.7	0.41	0.536	1.91
Vinyl	1	323	323.1	0.10	0.757	2.57
PP	1	2911	2911.1	0.92	0.363	1.95
GO	1	9430	9430.2	2.98	0.118	1.53
MS (SF)	1	47,606	47,606.0	15.04	0.004	1.32
C	1	7104	7103.8	2.24	0.168	1.27
PVA	1	17,164	17,164.1	5.42	0.045	1.45
Hybrid fiber use	1	1217	1216.8	0.38	0.551	3.26
Error	9	28,494	3166.0			
Total	17	102,137

### Compressive strength

This section contains different subsections to determine the effect of various parameters on the concrete compressive strength. Two curing periods of 7 and 28 days were considered for the experimental program.

#### Influence of recycled gloves

**Figure 15:**
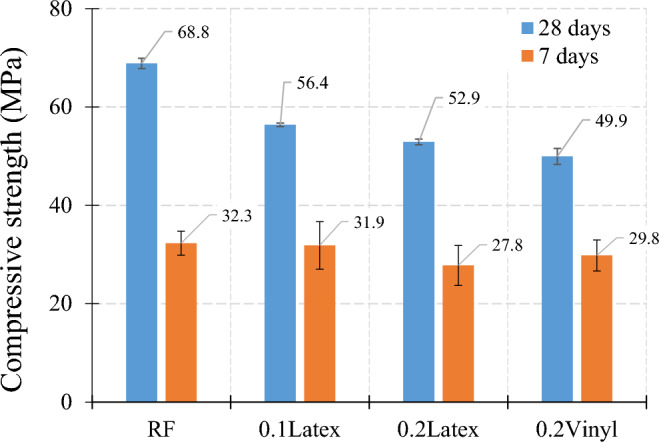
Influence of recycled gloves on compressive strength of mortar.

Figure 15 shows the influence of waste fibers on compressive strength. Findings revealed that using 0.1% and 0.2% latex fibers results in 18.0% and 23.1% decreases in compressive strength, respectively. Correspondingly, mixtures containing 0.2% vinyl fibers have − 27.5% lower strength as compared to the reference mixture. Also, experimental observations indicate that vinyl fiber has a higher degradative influence on compressive strength, as compared to latex fibers. Results show that strength reductions in 7-day curing samples are lower than in 28-day samples.

#### Influence of graphene oxide (GO)

**Figure 16:**
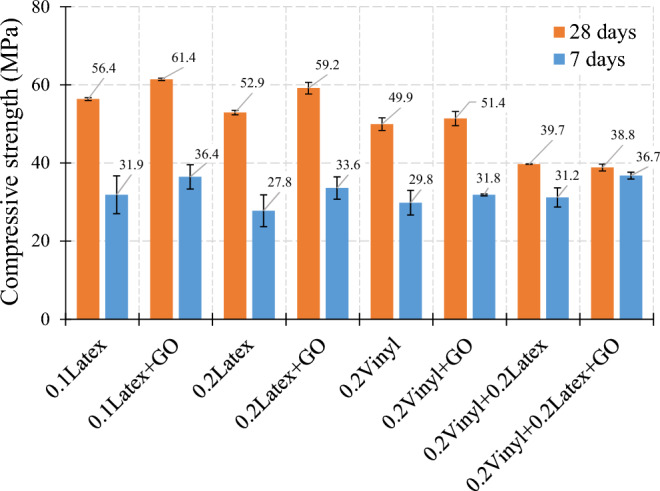
Influence of graphene oxide (GO) nanomaterials on compressive strength of mortar.

The impact of GO nanomaterials on the compressive strength is shown in Fig. 16. Generally, favourable results were obtained for using nanomaterials in mixtures containing waste disposable gloves. For instance, the addition of GO nanomaterials generates 8.9%, 11.9%, and 3.0% enhancements on the compressive strength of mixtures using 0.1% latex, 0.2% latex, and 0.2% vinyl, respectively, which was severally confirmed by the literature for normal cementitious composites^41^. However, results show that GO has no improvement in samples with hybrid fibers after a 28-day curing period. Moreover, results show that the rate of hydration was not efficient in the “0.2Latex + 0.2Vinyl” mixture so no considerable difference was recorded after 7 days compared to 28 days of curing.

#### Influence of polyvinyl alcohol (PVA)

**Figure 17:**
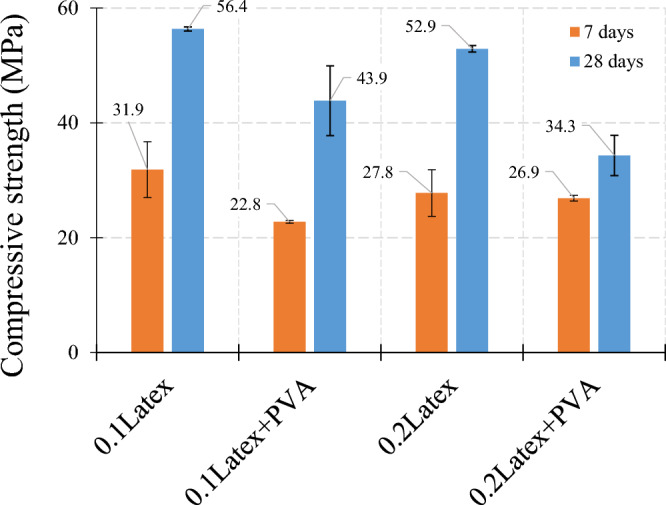
Influence of polyvinyl alcohol (PVA) on compressive strength of mortar.

The effect of PVA on the concrete compressive strength is shown in Fig. 17. Results show that using PVA in mixtures containing 0.1% latex results in a 28.5% and 22.2% decrease in compressive strength for samples with 7 days and 28 days curing, respectively. Regarding 0.2% latex fiber, results show that the addition of PVA causes 3.2% and 35.2 reductions in concrete compressive strength. Similar to reference mixtures, using PVA causes around + 18% improvement and a − 21.9% reduction in the compressive strength of samples within 7 days and 28 days of curing, respectively. Although the strength reduction of specimens using PVA is clearly confirmed by the present study, more experimental works should be considered in future research for the early-age strength of samples containing different dosages of PVA.

#### Influence of micro silica fume (SF)

**Figure 18:**
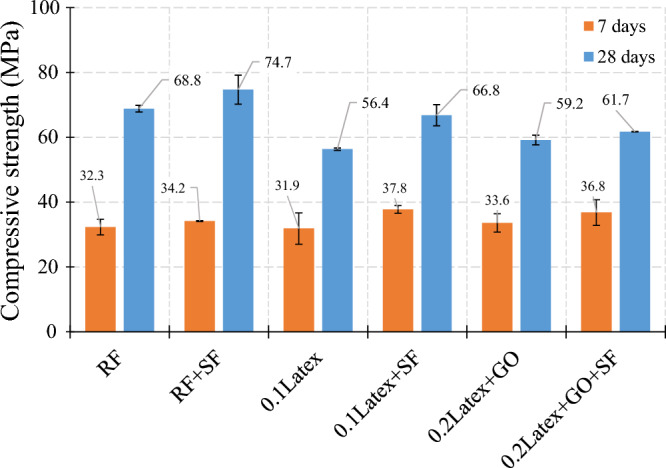
Influence of micro silica fume (SF) on compressive strength of mortar.

The effect of SF on the concrete compressive strength of mortars is shown in Fig. 18. For reference mixture, using SF causes an 8.6% increase in concrete compressive strength. In the case of samples containing 0.1% latex, the addition of SF results in an 18.4% increase in compressive strength. Similarly, a 4.2% improvement was recorded in concrete compressive strength in a mixture containing 0.2% latex. Generally, results clearly indicate that SF has a good synergistic effect with a low dosage of latex fibers (0.1%). A similar trend was observed for samples after 7 days of curing. In this case, 5.9%, 18.5%, and 9.5% improvements were recorded for “RF + SF”, “0.1Latex + SF”, and “0.2Latex + GO + SF”.

#### Influence of clay (C)

**Figure 19:**
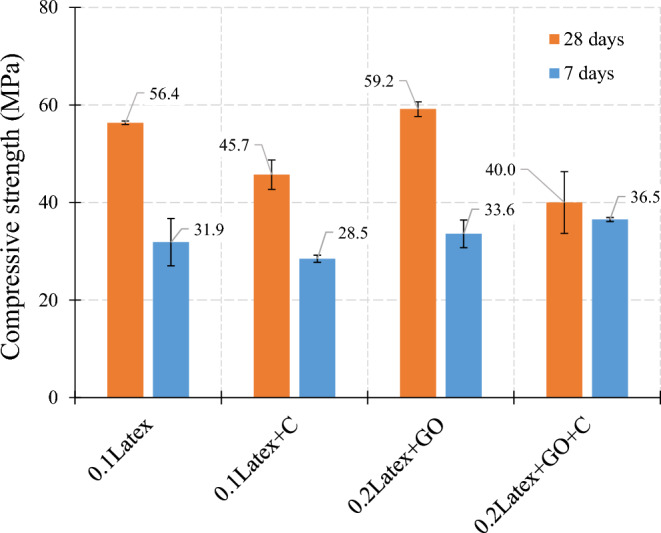
Influence of Cloisite 15A nanoclay (C) on compressive strength of mortar.

The effect of Cloisite 15A nanoclay (C) on the concrete compressive strength of mortars is shown in Fig. 19. Generally, results show that using Cloisite 15A nanoclay reduces the compressive strength of mortars. In the mixture with 0.1% latex fibers, using clay has − 19% and − 10.7% reductions after 7 days and 28 days of curing, respectively. Similarly, the addition of clay results in a − 32.4% reduction in the 28-day compressive strength of mortar. However, no clear trend was obtained for early-age strength. Hence, more future studies are necessary to concentrate on the early-age strength of mixtures containing Cloisite 15A nanoclay (C).

#### Influence of hybrid fibers

**Figure 20:**
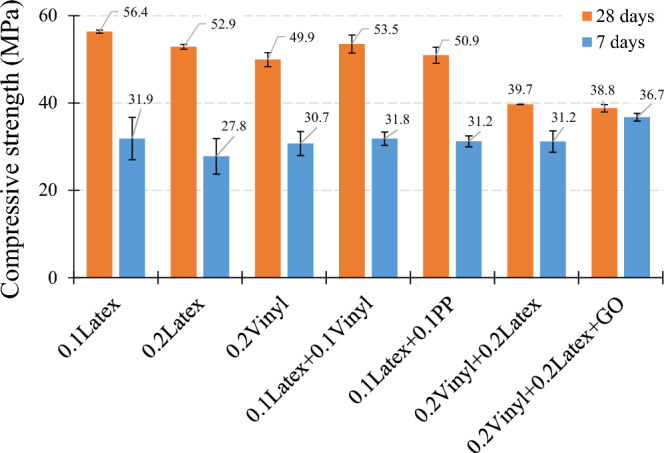
Influence of hybrid fibers on compressive strength of mortar.

The effect of hybrid fibers on the concrete compressive strength of mortars is shown in Fig. 20. Generally, results show that using a low dosage of hybrid fibers (“0.1Latex + 0.1fiber”) causes a comparable and higher 28-day compressive strength as compared to “0.2Latex” and “0.2Vinyl” mixtures. For instance, the “0.1Latex + 0.1Vinyl” mixture has a 1.13% higher compressive strength as compared to the “0.2Latex” mixture. However, considerable strength reductions were observed for a high dosage of hybrid fibers, so that − 25% and − 26.7% reductions were obtained for “0.2Latex + 0.2Vinyl” and “0.2Latex + 0.2Vinyl + GO”, respectively, as compared to “0.2Latex” mixture. However, more promising results were observed for 7-day samples such that 14.4%, 12.2%, 12.2%, and 32.2% strength improvements were recorded for “0.1Latex + 0.1Vinyl”, “0.1Latex + 0.1PP”, “0.2Latex + 0.2Vinyl”, and “0.2Latex + 0.2Vinyl + GO”, respectively.

#### Influence of printing layer delay

**Figure 21:**
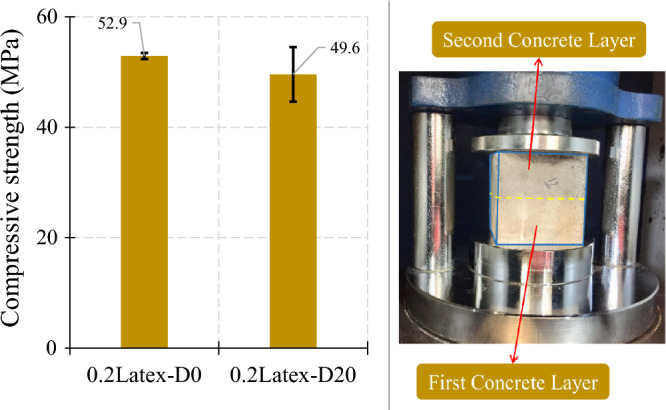
Influence of a delay in printing the second layer on compressive strength of mortar.

**Figure 22:**
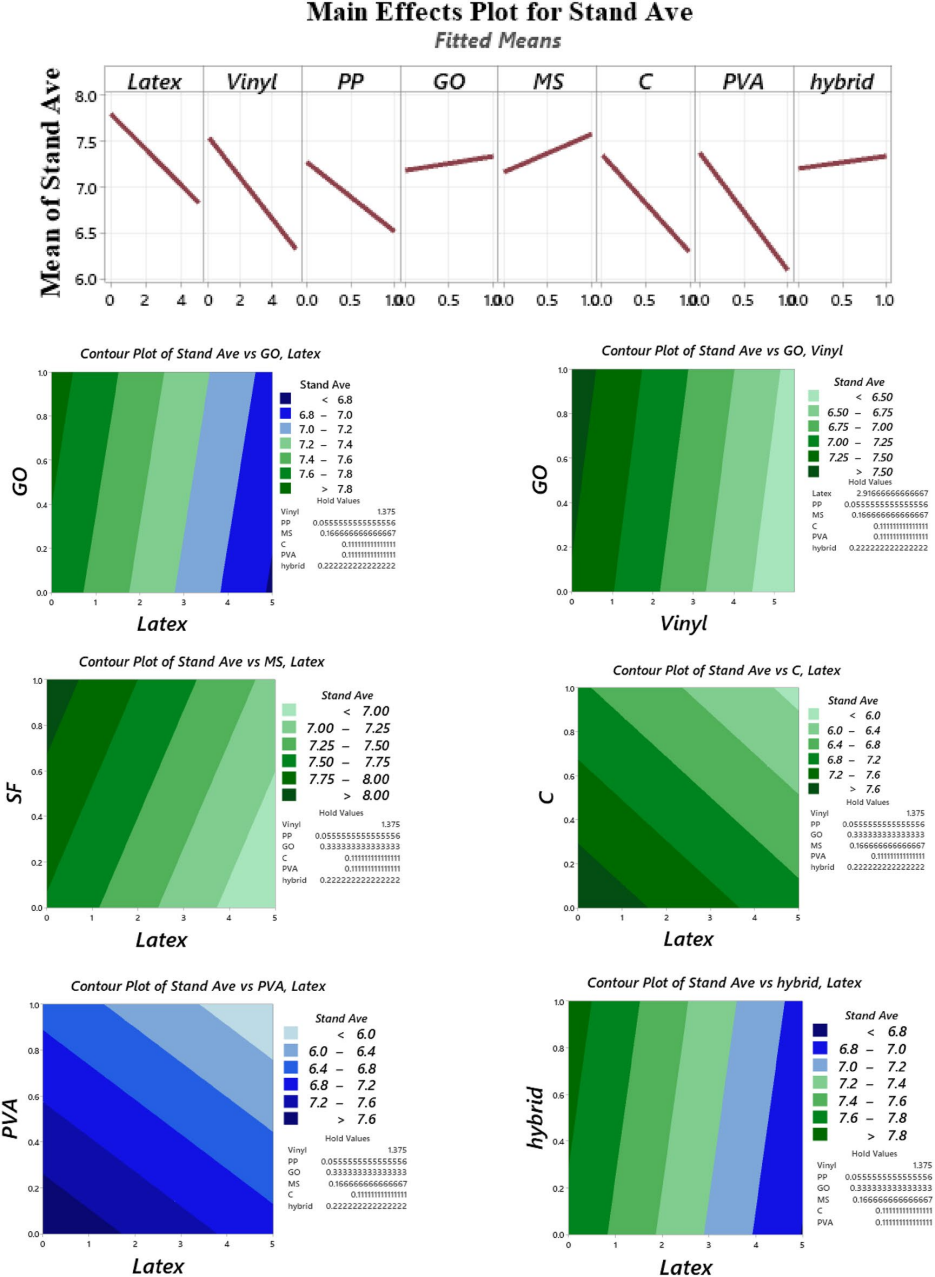
Analysis of variance (ANOVA) for 28 days of compressive strength results.

To have cohesive composite printed samples, the appropriate bond strength between layers should be provided. There should be a threshold for having a delay in printing layers in large-scale structures. Hence, a delay was considered for some cubic samples, in which the second layer was poured on top of the first layer after 20 min. Results show that a printing delay of 20 min results in a comparable and slightly lower reduction (− 6.2%) in the concrete compressive strength (Fig. 21). Hence, the delay between layers should be controlled and be lower than 20 min to maintain the appropriate mechanical characteristics. To comprehensively analyze the results, an ANOVA analysis was conducted on the results of compressive strength, which is shown in Fig. 22. Statistical results show that the addition of latex fiber, vinyl fiber, PP fiber, Cloisite 15A nanoclay (C), and PVA reduced the compressive strength while using GO nanomaterials, micro silica fume (SF or MS), and hybrid recycled fibers improved the compressive strength.

### Hardened density

**Figure 23:**
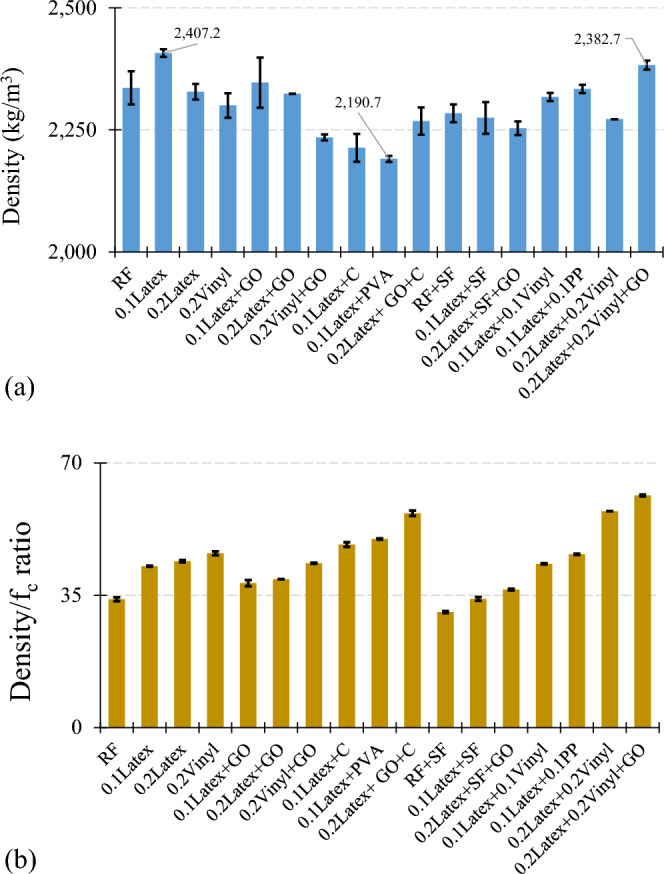
Effect of additives on the hardened density: (**a**) density of mixtures; (**b**) ratio of hardened density-to-compressive strength.

The hardened density of mixtures is illustrated in Fig. 23a. Results show that among different chemical and mineral additives, PVA causes a considerable reduction (− 8.9%) in the density of 3DCP containing 0.1% latex fibers. Moreover, results show that adding nanoclay and GO resulted in − 8.1% and − 2.5% reductions in the density of “0.1Latex” mixture. Using GO in “0.2Vinyl” caused a slight decrease of − 2.8% in the hardened density. Experimental findings also revealed that using SF in “0.1Latex” mixture reduced the density to − 5.5%. Using hybrid fibers also was efficient in lowering the hardened density so that mixtures of “0.1Latex + 0.1Vinyl” and “0.1Latex + 0.1PP” have − 3.8% and − 3.0% lower hardened density as compared to “0.1Latex” mixture. The ratio of hardened density-to-concrete compressive strength is shown in Fig. 23b. The results of this ratio indicate that mixtures of “RF + SF”, “0.1Latex + SF”, “0.2Latex + SF + GO”, and “0.1Latex + GO” have the lowest percentage among all mixtures. However, mixtures of “0.2Latex + 0.2Vinyl + GO”, “0.2Latex + 0.2Vinyl”, and “0.2Latex + GO + C” have the lowest density/f_c_ ratio, showing the weakest performance regarding the hardened density in relation with the mechanical strength.

### Tensile strength

**Figure 24:**
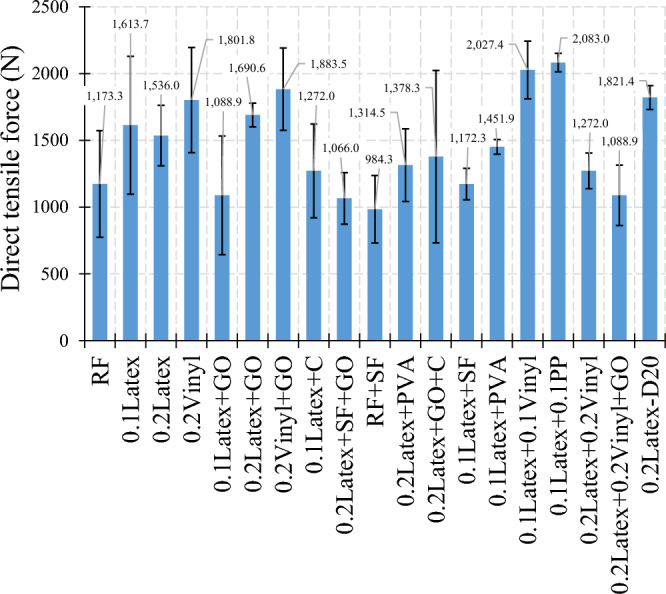
Results of direct tension test for all mixtures.

**Figure 25:**
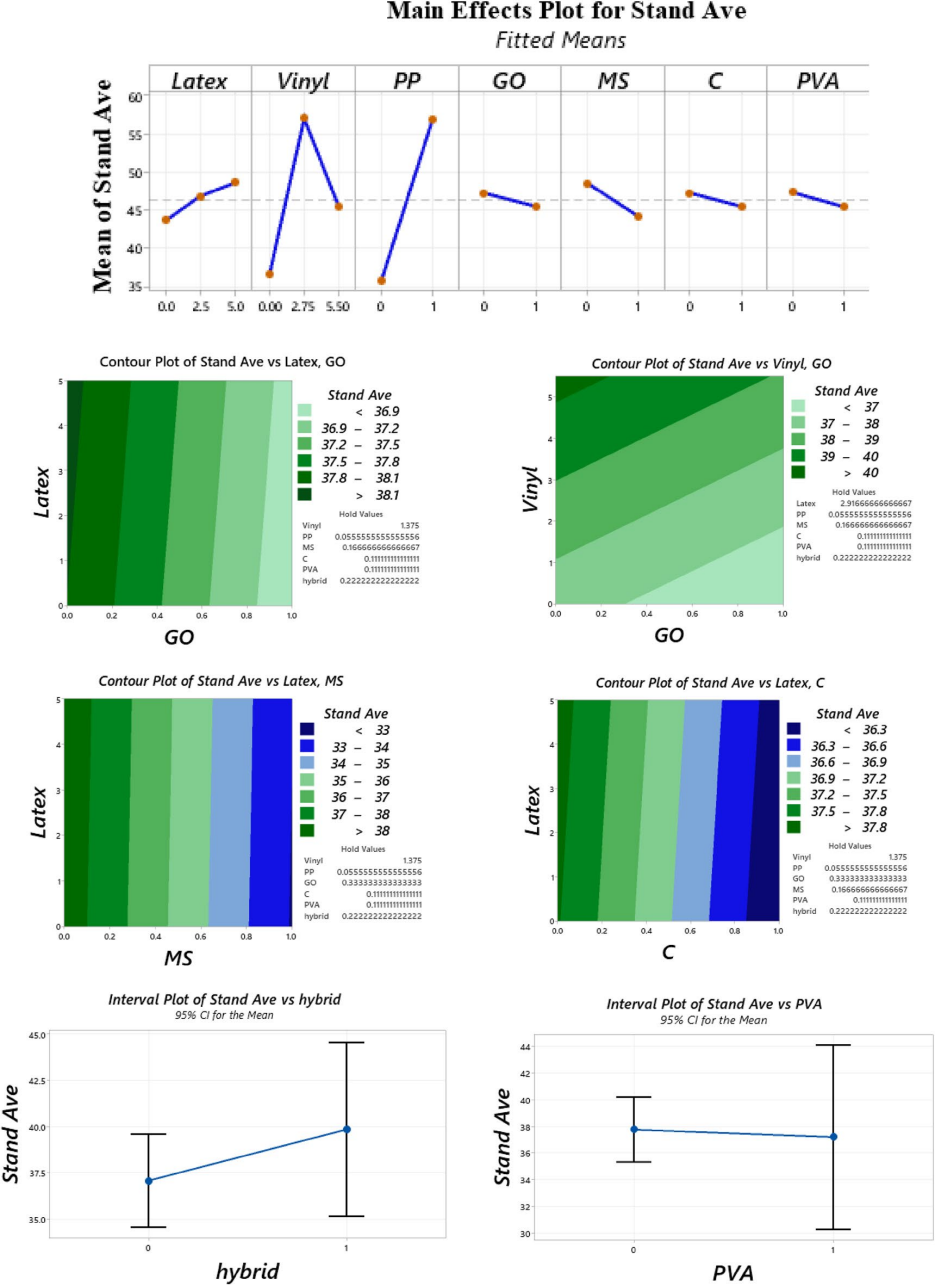
Analysis of variance (ANOVA) for direct tension test results.

The direct tensile strength results are illustrated in Fig. 24. Overall, findings indicate that using waste disposable gloves enhanced the tensile strength so that mixtures containing 0.1% and 0.2% latex fibers have 37.5% and 30.9% improvements in tensile strength, respectively. Furthermore, the addition of 0.2% vinyl fiber enhances the direct tensile strength by about 53.6%. Contrary to other admixtures, no precise trend was obtained for nanomaterials. For example, although no improvement in the direct tensile strength was observed for a GO-contained mixture containing 0.1% latex fibers, GO nanomaterials cause 10.1% and 4.5% enhancements in mixtures using 0.2% latex and 0.2% vinyl fibers, respectively. Moreover, experimental observations revealed that vinyl fiber has a higher impact on improving the direct tensile behaviour than latex fiber (Fig. 24). To comprehensively analyze the results of the direct tensile test, an ANOVA analysis was performed, which is shown in Table 4 and Fig. 25. Statistical analysis shows that PP fiber, vinyl fiber, and a hybrid combination of recycled fibers have the most effective among the studied parameters. Moreover, GO nanomaterials, SF, Cloisite 15A nanoclay (C), and PVA reduced the compressive strength. This reduction is more crucial for mixtures containing micro silica fume (SF or MS). Regarding hybrid fibers, results indicate that a lower dosage of fibers has a significant influence on tensile strength, while a higher dosage has comparable or slightly higher results. For instance, “0.1Latex + 0.1Vinyl” and “0.1Latex + 0.1PP” mixtures have 72.8% and 77.5% improvements in tensile strength, respectively. However, the “0.2Latex + 0.2Vinyl” mixture slightly (+ 8.4%) increased the tensile strength. Regarding mixtures containing GO nanomaterials, comparable and slightly lower results were obtained with a high SD. Hence, further experimental studies are needed for future works to extend the results of the present study.

**Table 4:** Analysis of variance (ANOVA) for direct tension results.

Source	DF	Adj SS	Adj MS	F-Value	P-Value	VIF
Latex	2	25.187	12.593	2.25	0.176	1.91
Vinyl	2	134.701	67.350	12.04	0.005	2.57
PP	1	141.962	141.962	25.38	0.002	1.95
GO	1	8.060	8.060	1.44	0.269	1.53
MS (SF)	1	34.970	34.970	6.25	0.041	1.32
C	1	4.396	4.396	0.79	0.405	1.27
PVA	1	4.310	4.310	0.77	0.409	1.45
Hybrid fiber use	1	92.868	92.868	16.60	0.005	3.26
Error	7	39.155	5.594			
Total	17	336.198

### Flexural strength

Two different types of samples were considered for the flexural test, including (1) plain samples without internal reinforcement; and (2) reinforced samples with metal welded wire mesh (WWM). Accordingly, two separate subsections are considered in this section to compare the results.

#### Specimens without internal reinforcement

**Figure 26:**
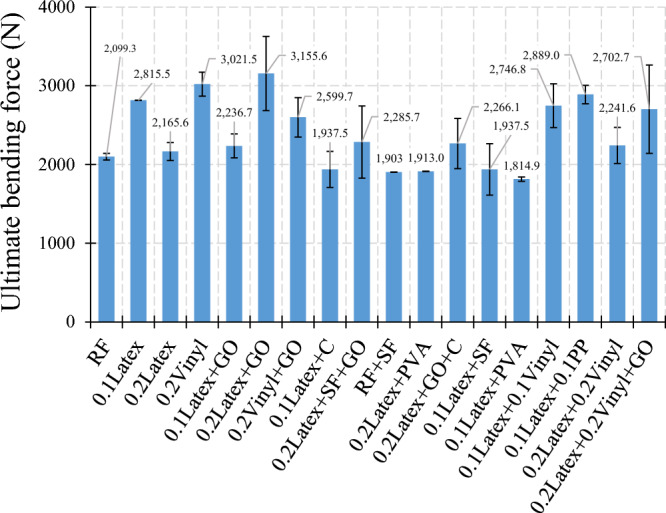
Results of flexural test for samples without internal reinforcement.

**Figure 27:**
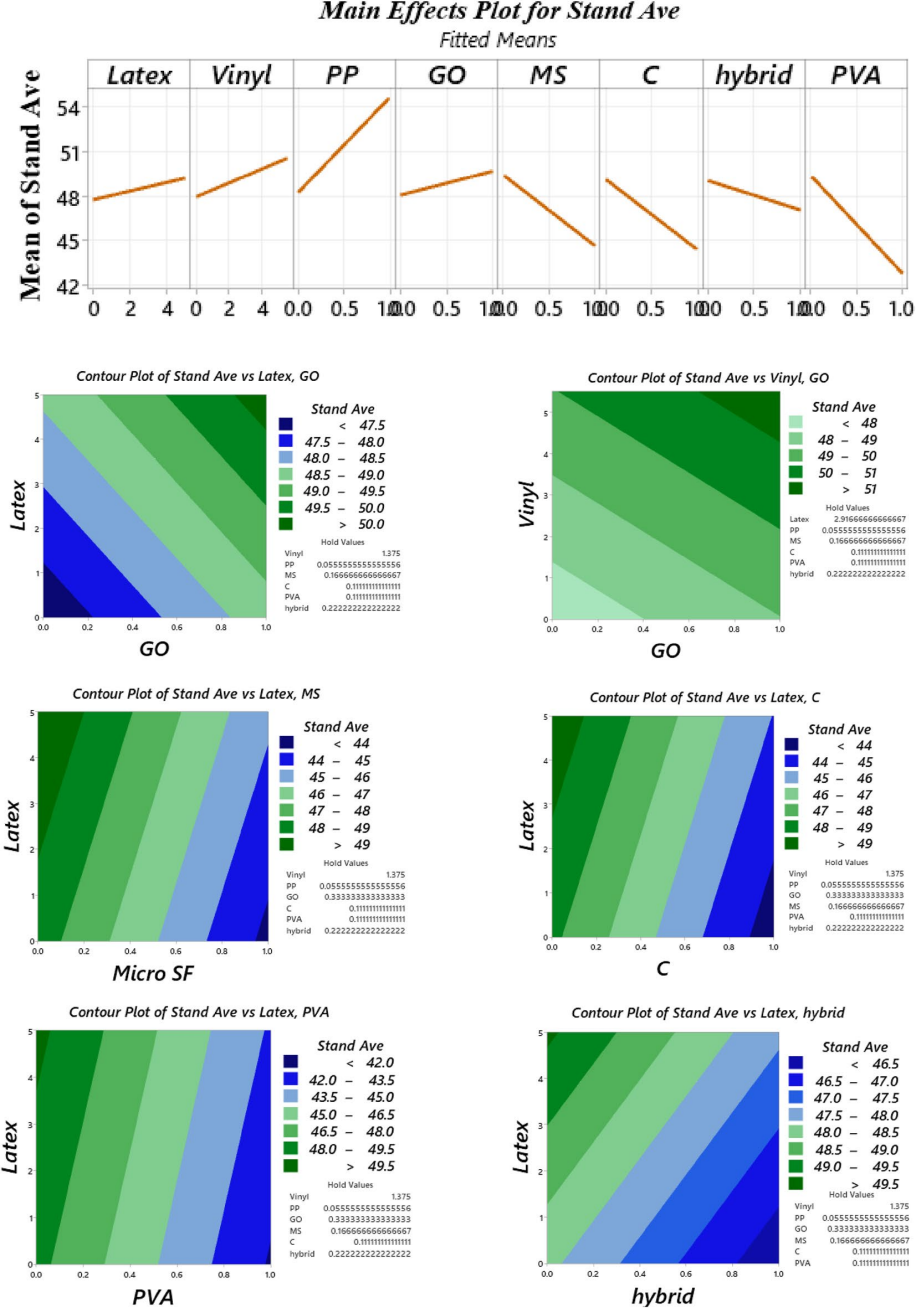
Analysis of variance (ANOVA) for flexural test results.

As illustrated in Fig. 6, lateral flexural loading was applied to the samples, in which two discrete layers of mortar were poured within the mould to simulate the 3DCP. The main point regarding this type of loading is to study the effect of bond strength between the layers with and without internal reinforcement. Results of the flexural test for all mixtures are shown in Fig. 26. Overall findings indicate that using waste disposable gloves increases the flexural strength of mixtures, so that the addition of 0.1% and 0.2% latex fibers causes a 17% and 1.7% enhancement in flexural strength, respectively. Lesser enhancement of flexural strength for mixtures containing 0.2% latex, compared to 0.1%, may be due to the greater compressive strength decrease in mixtures using a high amount of latex fibers. Regarding disposable vinyl gloves, findings show that the addition of 0.2% vinyl fibers results in a + 41.9% advance in flexural strength. No accurate tendency was obtained for GO-contained mixtures, which was correspondingly described by the literature for normal cementitious composites^43^. As illustrated in Fig. 26, experimental observations revealed that considering GO has no substantial impact on the flexural strength of latex fibers-contained mixtures, whereas using GO nanomaterials improves the bending force up to + 13.6%. To extensively explain the results, statistical analysis using ANOVA was performed in the present study, which is mentioned in Table 5 and Fig. 27. Results indicate that PP fiber and PVA have the most effect on the mixtures containing recycled fibers among the studied parameters. As shown in Fig. 27, latex fiber, vinyl fiber, PP fiber, and GO nanomaterials increased the flexural stiffness of samples, while silica fume, Cloisite 15A nanoclay (C), PVA, and hybrid fibers reduced the results.

**Table 5:** Analysis of variance (ANOVA) for flexural results.

Source	DF	Adj SS	Adj MS	F-value	P-value	VIF
Latex	2	15.350	7.675	0.65	0.551	1.91
Vinyl	2	38.153	19.077	1.61	0.265	2.57
PP	1	45.464	45.464	3.85	0.091	1.95
GO	1	5.500	5.500	0.47	0.517	1.53
MS (SF)	1	31.237	31.237	2.64	0.148	1.32
C	1	30.556	30.556	2.59	0.152	1.27
PVA	1	53.350	53.350	4.52	0.071	1.45
Hybrid fiber use	1	25.721	25.721	2.18	0.184	3.26
Error	7	82.694	11.813			
Total	17	319.258

#### Specimens with plain metal wire mesh

**Figure 28:**
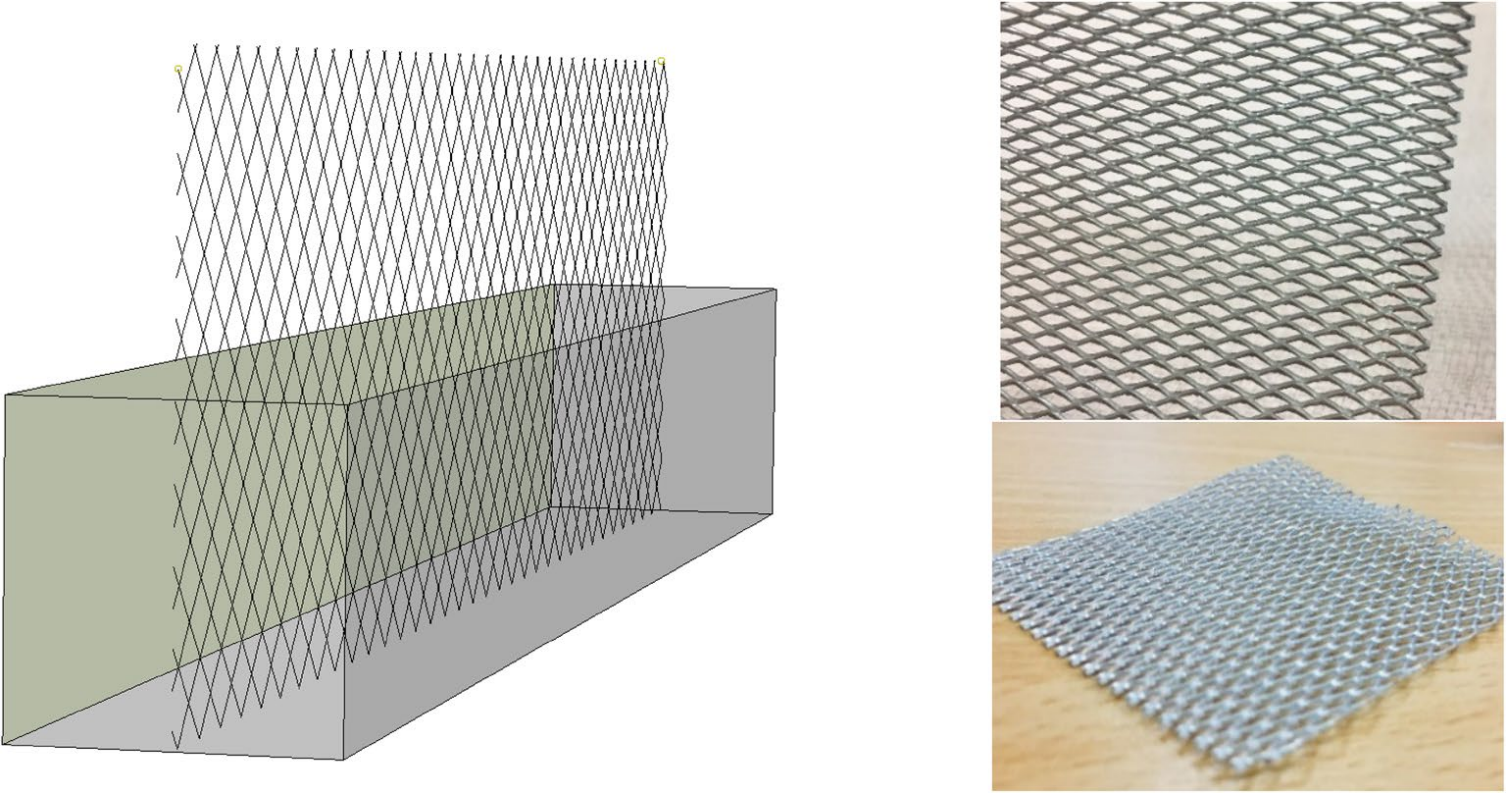
Metal welded wire mesh (WWM) considered in the experimental tests.

**Figure 29:**
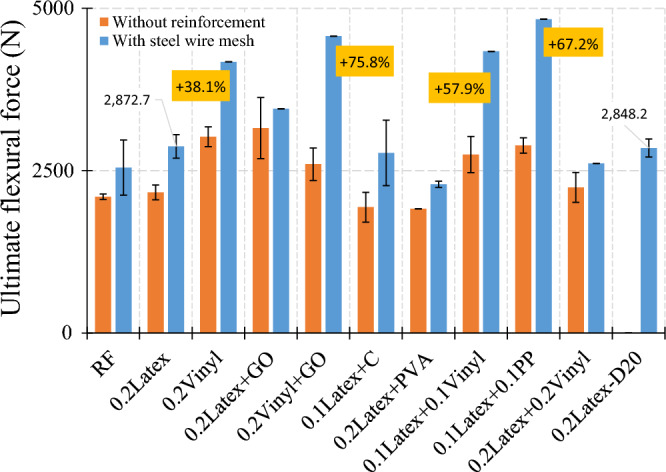
Effect of WWM on lateral ultimate flexural force.

**Figure 30:**
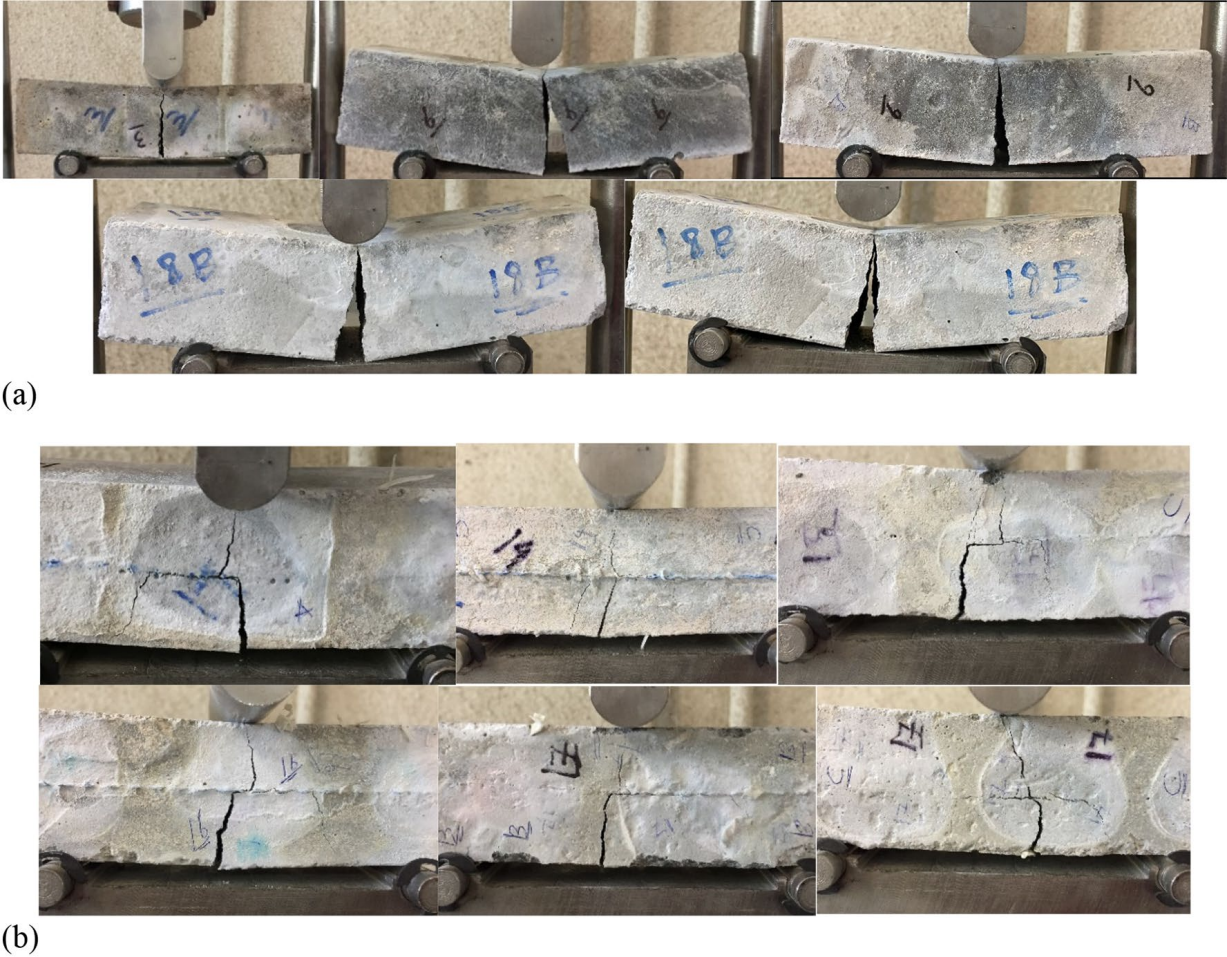
Failure mechanisms of samples after the flexural test: (**a**) brittle failure for samples without reinforcement; (**b**) ductile failure for samples with plain steel wire mesh.

As illustrated in Fig. 6, lateral flexural loading was applied to the samples, in which two separate layers of concrete were poured. Rhombus-shaped metal welded wire mesh (WWM) used in this study has a thickness of 0.50 mm and dimensions of 4.67 × 2.3 mm (Fig. 28). Also, yield stress, tensile strength, and relative elongation of WWM are 340 MPa, 500 MPa, and 18%, respectively. Some mixtures were used to determine the synergic effect of mixture composition and WWM on the lateral ultimate flexural stiffness of samples. As shown in Fig. 29, using WWM significantly improved the flexural stiffness of samples for all mixtures. Among studied mixtures, results show that “0.1Latex + 0.1PP”, “0.2Vinyl + GO”, “0.1Latex + 0.1Vinyl”, “0.2Vinyl” mixture showed the maximum ultimate force enhancement by + 67.2%, + 75.8%, + 57.9%, and + 38.1% improvements, respectively. Along with strength improvements, results also show that the failure modes of samples were also changed from brittle failure to ductile failure mode by using WWM internal reinforcement (Fig. 30). Regarding samples with WWM, results indicate that crack propagation modified in which crack path and widths are different before and after the WWM zone. Although the tensile face of the layered structures experienced large crack widths, no or very small crack width was observed at the top of the structures, showing that WWM was successful in absorbing the energy of the applied load along with the decentralization of tension. This can be efficient for reinforced 3D printed layers to be used in composite structures at the inner core between two steel plates (such as ships) to control the damages. The effect of the printing layer was also studied in this section in the reinforced sample with the “0.2Latex-D20” mixture. Results show that even with reinforcing samples, 20 min delay causes an − 11.3% reduction in ultimate flexural stiffness.

### CO_2_ emissions of mixtures

**Figure 31:**
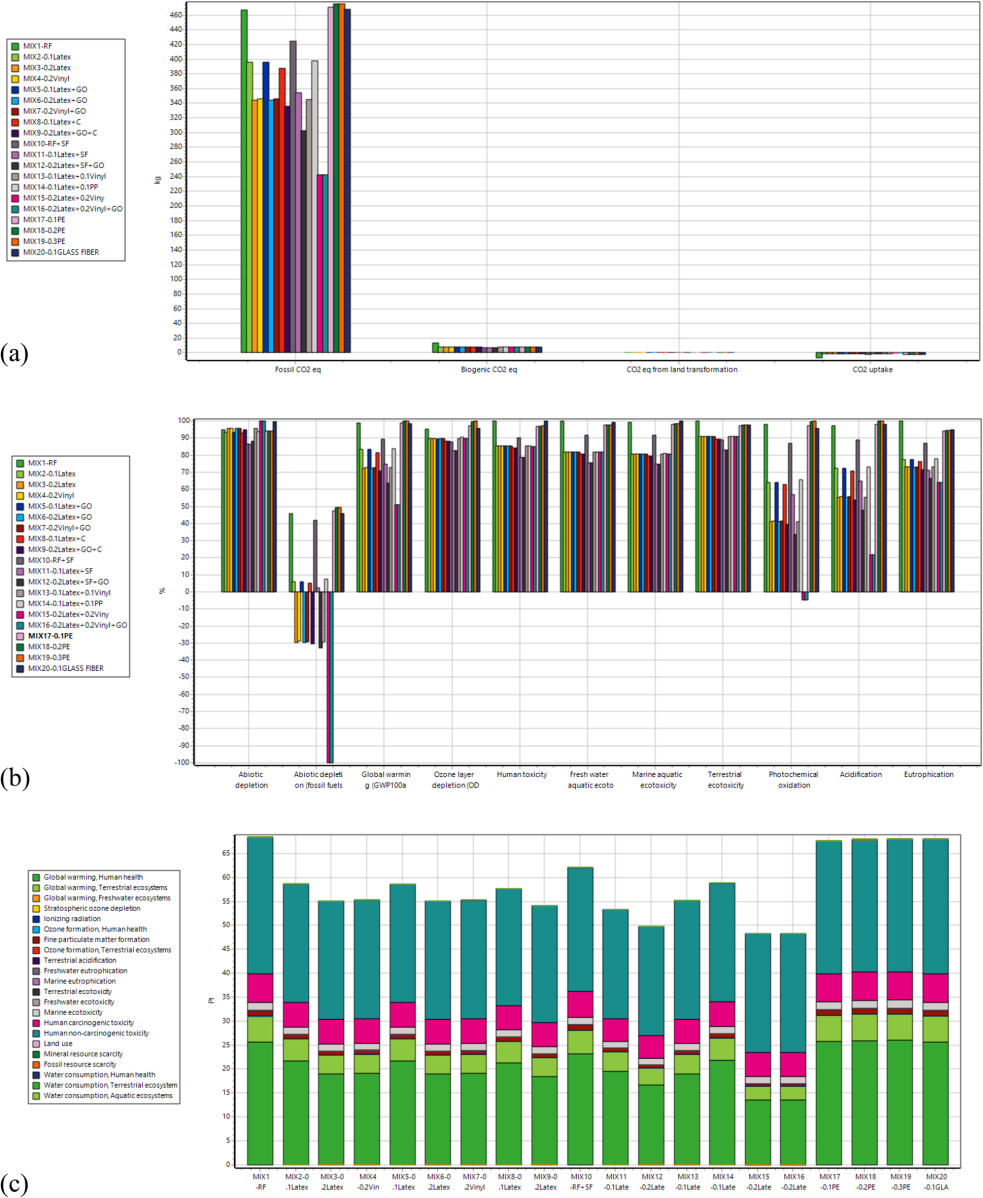
Carbon footprint (kg-CO_2_, eq) results of concrete mixtures containing different types of fibers: (**a**) GHG; (**b**) CML; (**c**) ReCiPe.

SimaPro software was used in the present study to measure the carbon footprint of mixtures. Different methods were employed in SimaPro software to determine the environmental impacts of a 3DCP mixture containing waste disposable gloves, including greenhouse gas (GHG), center of environmental science (CML), and ReCiPe 2016 methods. GHG environmental assessment corresponds to the extraction of raw materials, production or processing, transportation, use and end-of-life management of a product. CO_2_, eq indicates the quantity of CO_2_ that has the effect of global warming impacts. A measure of GHG can be described as CO_2_, eq, by multiplying the quantity of the GHG by its global warming potential (GWP) value. CML is a method considered to evaluate and calculate a product's environmental impact by employing several impact categories, such as eutrophication, ionization radiation, aquatic ecotoxicity, land use, and human toxicity. ReCiPe method contains both problem-oriented (midpoint) and damage-oriented (endpoint) impact categories, accessible for three different viewpoints of individualist, hierarchist, and egalitarian. At the midpoint level, 18 impact categories are considered in the ReCiPe method, while most of these midpoint impact classes are multiplied by damage factors at the endpoint level. Hence, these methods were considered in this section to determine the potential of tested mixtures for improving the sustainability of 3DCP. Carbon footprint (kg-CO_2_, eq/m^3^) results of concrete mixtures containing different types of fibers are shown in Fig. 31 based on CO_2_, eq in mass units (kg) and Global Warming Potential (GWP) content. To compare the results of mixtures containing waste disposable gloves as recycled fibers, other types of fibers were considered in SimaPro software, including polyethylene (PE) and glass fibers. Accordingly, twenty numbers of mixtures were tested by these methods. Generally, analysis using SimaPro software demonstrates that adding latex and vinyl recycled fibers within 3DCP mixtures causes the most significant promising environmental impact among other fibers. Using GHG analysis, the amount of CO_2_ emissions (kg) per functional unit mass of 3DCP mixtures (kg) is shown in Fig. 31a. It can be deduced from the results that 3DCP mixtures containing 0.1% and 0.2% latex fibers can reduce CO_2_ emissions by 15.2% and 26.3%, respectively, compared to the reference mixture. GHG analysis of the studied mixture indicates that 3DCP mixtures containing 0.2% latex and 0.2% vinyl fibers (0.4% recycled fibers) can reduce CO_2_ emissions up to 48.0%, showing a sustainable 3DCP mixture. Additionally, GHG analysis shows that recycled fibers cause more sustainable mixtures as compared to PE ones so that 3DCP mixtures containing 0.1% and 0.2% latex fibers have 15.7% and 27.6% lower CO_2_ emissions as compared to 3DCP mixtures containing 0.1% and 0.2% PE fibers respectively. Similarly, mixtures containing 0.1% glass fibers have 18.2% higher CO_2_ emissions than those containing 0.1% latex fibers. GHG analysis also reveals that SF admixture can cause a 12.2% reduction in CO_2_ emissions, among other additives, so the mixture of “0.2Latex + GO + SF” has 35.35 lower CO_2_ emissions than the reference mixture (RF). As shown in Fig. 31b,c, both CML and ReCiPe methods confirm the analysis of GHG, where mixtures containing 0.4% recycled fibers (0.2%Latex + 0.2%Vinyl) are the most sustainable 3DCP mixture among other mixtures containing other types and percentages of fibers. Accordingly, using recycled disposable gloves within 3DCP mixture not only improves some printing characteristics, but also considerably enhances sustainability by reducing CO_2_ emissions.

## Discussion

**Figure 32:**
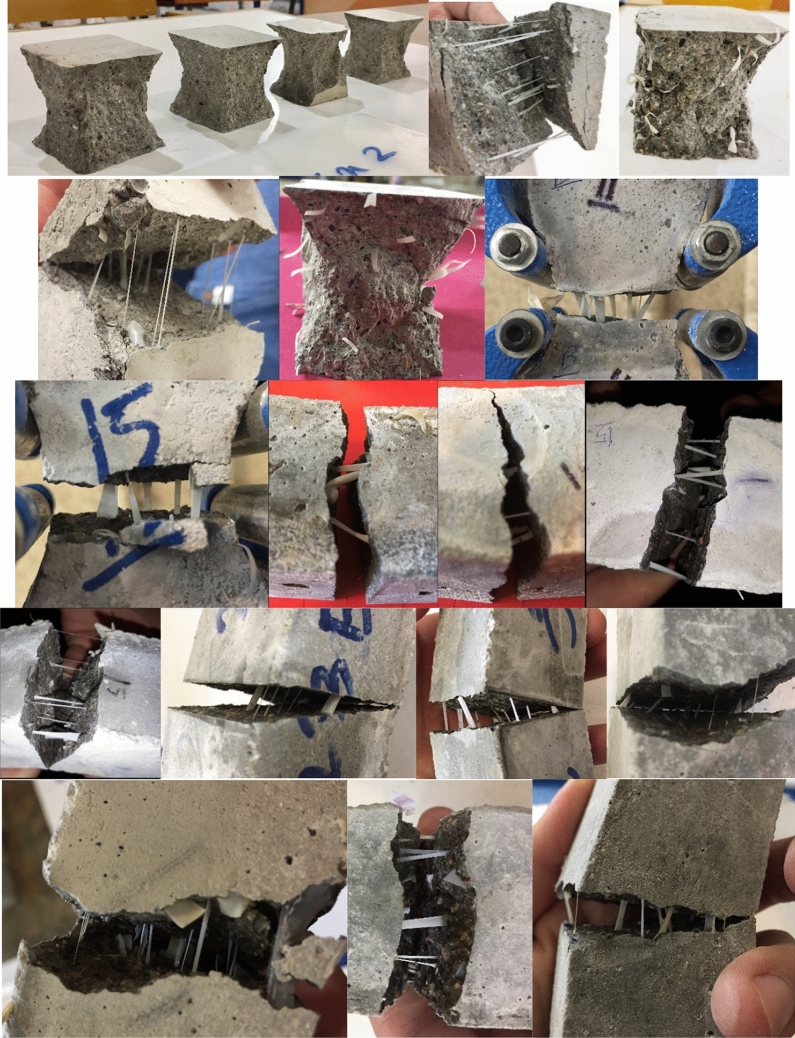
Failure mechanisms of samples after mechanical tests.

**Figure 33:**
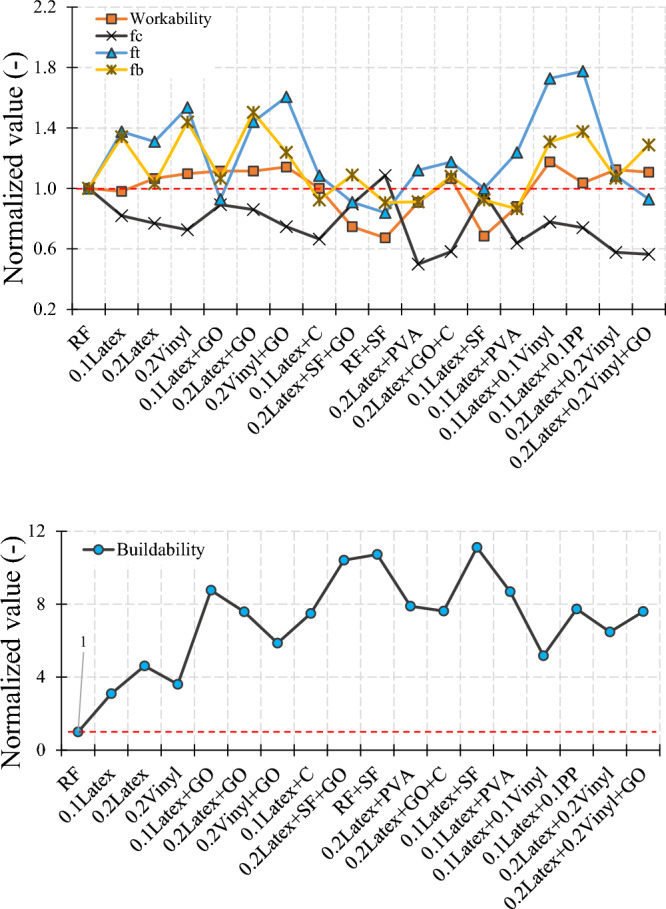
Summary of results by normalization to obtain the optimum mixture.

A summary of experimental findings is mentioned in Table 6. A summary of the failure modes of samples is also illustrated in Fig. 32. Results show that using recycled fibers can be an alternative approach as compared to commercial fibers for managing waste disposable gloves and improving the 3D printing characteristics of samples. However, it is worth mentioning that a specific technique is required to be presented in future works to reduce the size of the recycled fibers by providing a special production machine. To find the optimum mixture, this section also intends to summarize the results by normalizing the studied parameters, which is shown in Fig. 33. A standardized content higher than 1.0 indicates a suitable mixture with better performance than the RF mixture, while a value lower than 1.0 depicts unfavorable results. As illustrated before in Fig. 1, to attain an appropriate mixture considered for 3DCP, hardened and fresh features of the 3DCP mixture should be in good agreement. In other words, an optimal mixture for 3DCP needs to have adequate workability, enough buildability, satisfactory compressive strength, and appropriate layer bond strength. To accompany this methodology, standardizing or normalizing the considered factors can be efficient. As shown in Fig. 33, the combined impacts of disposable gloves and GO nanomaterials are beneficial so that the shape stability of the considered mixtures is meaningfully greater as compared to the reference mixture (RF). Overall, findings depict that the greatest mixtures trends found from the present investigation are 0.1Latex + GO > 0.2Latex + GO > 0.1Latex + 0.1Vinyl. However, as described before in Fig. 20, the compressive strength of the reference mixture (RF) is higher than all mixtures. Therefore, one scenario is to select the mixture with the minimum reduction in compressive strength of concrete, which is the “0.1Latex + GO” mixture. Another plan is to concentrate on the other factors. Taking into consideration the second plan, the “0.1Latex + 0.1Vinyl” mixture can be the best choice as the optimal mixture. Overall, results confirm that using different chemical and mineral admixtures is efficient to compensate for some deficiencies of latex- or vinyl-contained mixtures for consideration as a 3D printed mixture. However, it is worth noting that additional experimental investigations need to be considered for future research to approve the findings of the present work, and also develop the observations by producing large-scale 3DCP containing waste disposable gloves.

**Table 6:** Summary of obtained results.

Tests	Latex	Vinyl	GO	SF	C	PVA	Hybrid fiber
Fresh							
Workability	+	+	NA	−	−	−	
Buildability	+	+	+	+	+	+	+
Mechanical							
Hardened density	+	−	−	−	−	−	NA
Compressive test	−	−	+	+	−	−	+
Direct tension test	+	+	−	−	−	NA	+
Flexural test	+	+	+	−	−	−	−

Phrases of “+” and “−” show increasing and decreasing effects. “NA” denotes comparable results.

## Concluding remarks

Extensive experimental tests were carried out in the present work to study the opportunity of using waste disposable gloves as recycled fibers in cementitious composites for use in extrusion-based 3DCP specimens. Latex and vinyl waste gloves were used to generate recycled fibers with volume fractions of 0.1% and 0.2%. Also, different chemical and mineral admixtures were considered to improve the printability and mechanical characteristics of mixtures. Simplified experimental tests were considered to measure the 3D printing capacity of mixtures separately in two aspects of fresh and hardened characteristics. Based on the investigated observations, the following points are worth mentioning:

- Despite the commercial fibers, such as PP fiber, the addition of recycled fibers from waste latex and vinyl gloves improves the flowability of mixtures, and also increases the buildability and shape stability of mixtures. Moreover, using latex fibers results in a higher viscose mixture than vinyl fibers.
- Using GO is one of the main options to be used along with recycled fibers to control some deficiencies of mixtures containing recycled fibers. The addition of GO improved flexural, reduced tensile capacity, enhanced compressive strength, and developed fresh properties of latex- and vinyl-contained mixtures. Also, the hybrid uses of recycled fibers and GO meaningfully improved the shape stability of mixtures.
- Although using PVA causes considerable reductions in mechanical properties, significant improvement was observed for mixture buildability of 3DCP containing recycled latex and vinyl fibers.
- Sustainable 3DCP mixtures containing latex and vinyl fibers along with Cloisite 15A nanoclay had lower workability, considerably higher buildability, and lower mechanical strength. Hence, this clay material can be used to improve the cohesiveness of printed layers with recycled fibers.
- Using micro silica fume results in noticeably low workability and high buildability of 3DCP mixtures containing latex and vinyl fibers. Although higher compressive strength was observed in mixtures containing SF, lower tensile and flexural results were recorded. Hence, using a high dosage of recycled fibers is necessary for mixtures containing SF to compensate for the low tensile capacity.
- Hybrid use of latex and vinyl recycled fibers with low dosage (total 0.2%) showed promising results, in which the highest tensile stiffness was obtained. However, still, the compressive strength reduction of this mixture made some worries which can be compensated for by using SF.
- Considering internal reinforcement using WWM considerably improved the flexural stiffens of samples (up to + 75.8%) and also changed the brittle failure mode into ductile failure.
- Overall results show that “0.1Latex + GO” > “0.2Latex + GO” > “0.1Latex + 0.1Vinyl” are the optimum compositions among the studied mixtures. Also, mixtures containing 0.4% recycled fibers (0.2%Latex + 0.2%Vinyl) are the most sustainable 3DCP mixture among other mixtures.

It is worth mentioning that more experimental and analytical studies are required for future works to extend the present study's findings and investigate environmental aspects of using disposable waste gloves as recycled fibers in sustainable 3DCP, such as evaluating the CO_2_ emission and life cycle assessment. Moreover, only workability and buildability tests were considered in the peent study, while specific research is necessary for future investigation on the rheology of the proposed sustainable mixtures. Furthermore, as recycled disposable gloves contain antibacterial or moisturizing agents, a practical approach is required to clean these recycled latex and vinyl gloves and convert them into sustainable fibers, which needs to be discussed in future studies.

## Data Availability

All data generated and analyzed during this study are included in this paper.

## References

[1] Jędruchniewicz K, Ok YS, Oleszczuk P (2021). COVID-19 discarded disposable gloves as a source and a vector of pollutants in the environment. J. Hazard. Mater..

[2] Marandi, R. The necessity of crump tires recycling in urban management. *In Proceedings of the 2nd National Conference on Solid Waste Management and its Role in Urban Planning* (Tehran, Iran, 2005).

[3] Shah AA, Hasan F, Shah Z, Kanwal N, Zeb S (2013). Biodegradation of natural and synthetic rubbers: A review. Int. Biodeterior. Biodegrad..

[4] Nuzaimah M, Sapuan S, Nadlene R, Jawaid M (2018). IOP Conference Series: Materials Science and Engineering.

[5] Shu X, Huang B (2014). Recycling of waste tire rubber in asphalt and portland cement concrete: An overview. Constr. Build. Mater..

[6] Riyajan S-A, Intharit I, Tangboriboonrat P (2012). Physical properties of polymer composite: Natural rubber glove waste/polystyrene foam waste/cellulose. Ind. Crops Prod..

[7] Misman MA, Azura AR (2020). Advanced Materials Research.

[8] Javali S (2017). Eco-concrete for sustainability: Utilizing aluminium dross and iron slag as partial replacement materials. Clean Technol. Environ. Policy.

[9] Shahbazpanahi S, Manie S, Faraj RH, Seraji M (2021). Feasibility study on the use of tagouk ash as pozzolanic material in concrete. Clean Technol. Environ. Policy.

[10] Eldin NN, Senouci AB (1993). Rubber-tire particles as concrete aggregate. J. Mater. Civ. Eng..

[11] Aslani F (2016). Mechanical properties of waste tire rubber concrete. J. Mater. Civ. Eng..

[12] Aslani F, Deghani A, Asif Z (2020). Development of lightweight rubberized geopolymer concrete by using polystyrene and recycled crumb-rubber aggregates. J. Mater. Civ. Eng..

[13] Naito C, States J, Jackson C, Bewick B (2014). Assessment of crumb rubber concrete for flexural structural members. J. Mater. Civ. Eng..

[14] Zhang Y, Zhao Z (2015). Internal stress development and fatigue performance of normal and crumb rubber concrete. J. Mater. Civ. Eng..

[15] PashangPisheh Y, Mir Mohammad Hosseini M (2019). Experimental investigation of mechanical behavior of plastic concrete in cutoff walls. J. Mater. Civ. Eng..

[16] PashangPisheh Y, Mir Mohammad Hosseini M (2020). Laboratory study on the cyclic and postcyclic behavior of plastic concrete used in cutoff walls of embankment dams. J. Mater. Civ. Eng..

[17] Liu F, Yan Y, Li L, Lan C, Chen G (2015). Performance of recycled plastic-based concrete. J. Mater. Civ. Eng..

[18] ALbiajawi, M. I. *et al.* Performance of sustainable concrete containing recycled latex gloves and silicone catheter under elevated temperature. *J. King Saud Univ. Eng. Sci.* (2021).

[19] Mousavi SS, Dehestani M (2022). Influence of latex and vinyl disposable gloves as recycled fibers in 3D printing sustainable mortars. Sustainability.

[20] Kilmartin-Lynch S, Roychand R, Saberian M, Li J, Zhang G (2022). Application of COVID-19 single-use shredded nitrile gloves in structural concrete: Case study from Australia. Sci. Total Environ..

[21] Ismail H, Galpaya D, Ahmad Z (2009). The compatibilizing effect of epoxy resin (EP) on polypropylene (PP)/recycled acrylonitrile butadiene rubber (NBRr) blends. Polym. Test..

[22] Mathew G, Singh R, Nair N, Thomas S (2001). Recycling of natural rubber latex waste and its interaction in epoxidised natural rubber. Polymer.

[23] Mathew G, Nah C, Rhee J, Singh R (2006). Multifiller-matrix adhesion effects in epoxidized natural rubber. J. Elastomers Plast..

[24] Khaloo AR, Dehestani M, Rahmatabadi P (2008). Mechanical properties of concrete containing a high volume of tire–rubber particles. Waste Manage..

[25] Dehestani M, Khaloo AR, Rahmatabadi P (2008). Investigation of the behavior of rubberized concrete under uniaxial compressive test. J. Adv. Mater. Eng. (Esteghlal).

[26] Mousavi SS, Dehestani M (2022). Structures.

[27] Hussein HH, Edan O, Ahmed MK (2017). Mechanical, thermal and acoustical properties of concrete with fine polyvinyl chloride (PVC). Iraqi J. Civ. Eng..

[28] Bolat H, Erkus P (2016). Use of polyvinyl chloride (PVC) powder and granules as aggregate replacement in concrete mixtures. Sci. Eng. Compos. Mater..

[29] Mohammed AA, Mohammed II, Mohammed SA (2019). Some properties of concrete with plastic aggregate derived from shredded PVC sheets. Constr. Build. Mater..

[30] Merlo A, Lavagna L, Suarez-Riera D, Pavese M (2020). Mechanical properties of mortar containing waste plastic (PVC) as aggregate partial replacement. Case Stud. Constr. Mater..

[31] Mohammed, N. S., Hamza, B. A., AL-Shareef, N. A. H. & Hussein, H. H. Structural behavior of reinforced concrete slabs containing fine waste aggregates of polyvinyl chloride. *Buildings* **11**(1), 26.10.3390/buildings11010026 (2021).

[32] Senhadji Y (2015). Effect of incorporating PVC waste as aggregate on the physical, mechanical, and chloride ion penetration behavior of concrete. J. Adhes. Sci. Technol..

[33] Mortazavi, S. B., Rasoulzadeh, Y., Yousefi, A. A. & Khavanin, A. Properties of modified bitumen obtained from vacuum bottom by adding recycled waste polymers and natural bitumen (2010).

[34] Bhattacherjee S (2021). Sustainable materials for 3D concrete printing. Cement Concr. Compos..

[35] Dey D, Srinivas D, Panda B, Suraneni P, Sitharam T (2022). Use of industrial waste materials for 3D printing of sustainable concrete: A review. J. Clean. Prod..

[36] Qasimi MA, Zulayq DMA, Seifan M (2020). Mechanical and rheological properties of 3D printable cement composites. Recent Prog. Mater..

[37] Gislason S (2022). Porous 3D printed concrete beams show an environmental promise: A cradle-to-grave comparative life cycle assessment. Clean Technol. Environ. Policy.

[38] Chantrelle, C., Mousavi, S. & Ouellet-Plamondon, C. Effect of chemical admixtures on rheology, hydration and strength (2017).

[39] Lin A, Tan YK, Wang C-H, Kua HW, Taylor H (2020). Utilization of waste materials in a novel mortar–polymer laminar composite to be applied in construction 3D-printing. Compos. Struct..

[40] Bai G, Wang L, Ma G, Sanjayan J, Bai M (2021). 3D printing eco-friendly concrete containing under-utilised and waste solids as aggregates. Cement Concr. Compos..

[41] Mousavi SS, Mousavi Ajarostaghi SS, Bhojaraju C (2020). A critical review of the effect of concrete composition on rebar–concrete interface (RCI) bond strength: A case study of nanoparticles. SN Appl. Sci..

[42] Mousavi SS, Guizani L, Bhojaraju C, Ouellet-Plamondon C (2021). The effect of air-entraining admixture and superabsorbent polymer on bond behaviour of steel rebar in pre-cracked and self-healed concrete. Constr. Build. Mater..

[43] Bhojaraju C, Mousavi SS, Brial V, DiMare M, Ouellet-Plamondon CM (2021). Fresh and hardened properties of GGBS-contained cementitious composites using graphene and graphene oxide. Constr. Build. Mater..

[44] ASTM International (2020). ASTM C1437.

[45] Falliano D, De Domenico D, Ricciardi G, Gugliandolo E (2020). 3D-printable lightweight foamed concrete and comparison with classical foamed concrete in terms of fresh state properties and mechanical strength. Constr. Build. Mater..

[46] Pilehvar S, Arnhof M, Pamies R, Valentini L, Kjøniksen A-L (2020). Utilization of urea as an accessible superplasticizer on the moon for lunar geopolymer mixtures. J. Clean. Prod..

[47] Abudawaba F, Gomaa E, Gheni A, ElGawady M (2022). Developing mix proportions for class C fly ash-based alkali-activated 3D-printed concrete mixtures. Transp. Res. Rec..

[48] International, A. in *ASTM C190* (1990).

[49] Ye J, Cui C, Yu J, Yu K, Dong F (2021). Effect of polyethylene fiber content on workability and mechanical-anisotropic properties of 3D printed ultra-high ductile concrete. Constr. Build. Mater..

[50] Bhojaraju C, Mousavi SS, Ouellet-Plamondon CM (2022). Influence of GGBFS on corrosion resistance of cementitious composites containing graphene and graphene oxide. Cement Concr. Compos..

[51] Minitab, I. MINITAB statistical software. *Minitab Release* **13** (2000).

[52] Srinivas D, Dey D, Panda B, Sitharam TG (2022). Printability, thermal and compressive strength properties of cementitious materials: A comparative study with silica fume and limestone. Materials.

[53] Panda B, Ruan S, Unluer C, Tan MJ (2019). Improving the 3D printability of high volume fly ash mixtures via the use of nano attapulgite clay. Compos. B Eng..

